# Reframing Porous Carbon as a Transport Framework for High‐Rate CO_2_ Electroreduction

**DOI:** 10.1002/advs.78727

**Published:** 2026-11-10

**Authors:** Hyunwook Kim, Eunsol Kang, Subin Park, Hyeonggeon Lee, Jun Woo Park, Dongjin Kim, Kezia Langie, Younghyun Chae, Nara Lee, Dong‐Joon Lee, Ki Bong Lee, Ung Lee, Da Hye Won

**Affiliations:** ^1^ Clean Energy Research Center Korea Institute of Science and Technology Seoul Republic of Korea; ^2^ Department of Chemical Engineering Queen's University Kingston Ontario Canada; ^3^ Department of Chemical and Biological Engineering Korea University Seoul Republic of Korea; ^4^ Department of Chemistry Kookmin University Seoul Republic of Korea; ^5^ Department of Chemical and Biomolecular Engineering Sogang University Seoul Republic of Korea; ^6^ Division of Energy and Environmental Technology, KIST School University of Science and Technology (UST) Seoul Republic of Korea

**Keywords:** carbon support, CO_2_ electroreduction, computational fluid dynamics, mass transport, porosity

## Abstract

Porous carbons are widely used as supports for Cu‐based CO_2_ reduction electrodes, yet how their pore structures regulate reactant transport within the catalyst layer remains insufficiently understood. Here, carbon blacks with distinct hierarchical textural characteristics are systematically investigated, revealing a consistent association between more developed pore architecture and higher C_2_H_4_ productivity. Guided by this trend, a hierarchically integrated Cu/C catalyst is designed by forming Cu nanodomains throughout the carbon framework and compared with a benchmark prepared by physically mixing commercial Cu nanoparticles with the same carbon support. The integrated Cu/K6 catalyst achieves a C_2_H_4_ partial current density of 355 mA cm^–^^2^ with a C_2_H_4_ Faradaic efficiency of 50.7% at a total current density of 700 mA cm^−2^. Under these conditions, Cu/K6 exhibits a cathode potential approximately 0.3 V less negative and a 20% higher C_2_H_4_ partial current density than the physically mixed benchmark. Mixed kinetic‐transport analysis indicates that the integrated architecture shifts the apparent transport‐limitation threshold to higher current densities. Multiphysics computational fluid dynamics simulations further reveal shorter effective CO_2_ diffusion paths and mitigated local CO_2_ depletion. These findings establish hierarchical Cu–carbon integration as an effective strategy for alleviating CO_2_ transport limitations and enhancing C_2_H_4_ production.

## Graphical abstract

Hierarchical integration of Cu nanodomains with porous carbon frameworks promotes reactant transport during high‐rate CO_2_ electroreduction. Compared with a physically mixed benchmark, the integrated architecture shortens effective transport paths, mitigates local CO_2_ depletion, and lowers cathodic overpotential, enabling higher ethylene Faradaic efficiency and partial current density. Multiphysics simulations provide a consistent mechanistic interpretation of these transport benefits.

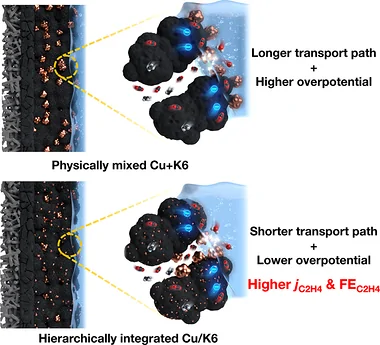

## 1 Introduction

Porous carbon materials have been extensively employed as supports for metal‐based catalysts in electrochemical CO_2_ reduction reaction (CO_2_RR) electrodes [1, 2]. They are considered to enhance catalyst stability, metal dispersion, and electrical conductivity [3], while their characteristic pore structures are thought to influence wetting behavior, reactant accessibility, and mass transport within the catalyst layer [4, 5]. In practice, carbon is typically introduced either by co‐mixing with the catalyst ink [6, 7] or by depositing a carbon overlayer on the catalyst [8, 9]; both approaches have proven effective in improving structural integrity, maintaining electrical connectivity, and increasing surface hydrophobicity to mitigate flooding. In this regard, carbon black is more frequently adopted than graphitic carbons or carbon nanotubes, as its interconnected pore network and tunable textural properties tend to deliver more favorable CO_2_RR outcomes [10, 11].

To gain deeper insight into the functional role of carbon black, Cu‐based systems capable of producing both C_1_ and C_2_ products have provided an effective platform for investigating how the carbon environment shapes reaction pathways and product distribution. In conventional Cu–carbon black configurations, carbon primarily acts as a conductive scaffold that facilitates charge transport and stabilizes the catalyst structure. When configured as an overlayer, it additionally serves as a protective layer that prevents Cu oxidation and preserves active metallic sites [8], whereas in oxide‐derived Cu on mesoporous carbon, the porous matrix stabilizes *CO intermediates and promotes C_2_H_4_ formation [12]. Beyond these structural and electronic roles, carbon black can regulate the catalyst‐layer microenvironment through its interconnected pore network and surface wetting characteristics, thereby influencing electrolyte intrusion, gas‐phase reactant accessibility, and the local availability of CO_2_‐derived intermediates. Recent findings support this approach, showing that higher CO_2_ availability near Cu surfaces stabilizes key intermediates and promotes C_2_H_4_ formation through the generation of Cu^δ+^‐OH species under CO_2_‐rich conditions that favor C─C coupling [13].

Building on these observations, identifying how the structural characteristics of carbon black regulate the local reaction environment is essential for effectively leveraging its advantages in CO_2_RR electrodes. In particular, the hierarchical micro/mesopore network constitutes the fundamental framework of carbon black, governing how gases and liquids access active sites and how reaction intermediates are distributed within the catalyst layer [14, 15, 16]. These textural characteristics can influence phase distribution and mass transport at the gas−liquid−solid interface and, in turn, modulate local CO_2_ availability and *CO coverage [17]. Nevertheless, pore architecture is inherently coupled with other support‐dependent properties, including wettability, electrical connectivity, surface chemistry, and aggregate morphology. Its contribution is therefore difficult to isolate as a single material variable, leaving the relationship between carbon architecture, the local reaction environment, and CO_2_RR performance insufficiently understood.

In this study, we address this gap by screening commercial carbon blacks with distinct hierarchical pore characteristics and examining how carbon pore structure influences CO_2_‐to‐C_2_H_4_ conversion in Cu‐based cathodes. Guided by the observed structure–performance trend, we designed a hierarchically integrated Cu–carbon architecture in which Cu nanodomains are distributed throughout the carbon framework. This architecture was compared with a practical benchmark prepared by physically mixing commercial Cu nanoparticles with the same carbon support. This comparison aims to determine whether closer integration of Cu sites with the carbon pore network, which is expected to shorten effective CO_2_ diffusion distances and increase the spatial overlap between CO_2_ transport pathways and catalytic sites, enhances C_2_H_4_ production. Computational fluid dynamics (CFD) simulations were employed to rationalize how the local transport environments created by the different Cu–carbon architectures influence C_2_H_4_ production, thereby linking carbon pore architecture with transport‐associated CO_2_RR performance [18].

## 2 Results and Discussion

### 2.1 Carbon Loading and Support Pore Architecture

Prior studies that coupled Cu catalysts with porous carbons report performance gains across a broad range of carbon loadings and pore structures. Because carbon can simultaneously influence electrical conductivity, wettability, and mass transport, however, the effect of carbon content itself is rarely discussed independently [5, 19, 20]. Across these reports, performance trends consistently point to two patterns: an empirical optimum in the carbon‐to‐Cu ratio and a dependence on textural features of the carbon support, suggesting that pore architecture is a critical design variable that can contribute alongside conductivity and wettability. However, whether carbon loading alone consistently alters CO_2_RR performance has not been explicitly verified. Therefore, as an initial step, we examined the effect of carbon loading on CO_2_RR performance by varying the amount of porous carbon black (Ketjen Black EC‐600JD, K6) physically mixed with commercial Cu nanoparticles from 0 to 30 wt.% (Figure 1a). These measurements were performed in a zero‐gap membrane electrode assembly (MEA), a configuration that minimizes ohmic losses and sustains continuous CO_2_ delivery to the cathode, providing a robust platform for CO_2_RR performance benchmarking (Figure S1). Under CO_2_RR conditions where C_2+_ production dominates (−3.2 V to −3.5 V), even modest carbon loadings had a pronounced effect on both current density and selectivity, underscoring that carbon loading is a meaningful electrode design variable in this regime. Relative to the carbon‐free Cu cathode, K6 addition influenced both the C_2_H_4_ FE and partial current density (*j*_C2H4_), but the extent of improvement varied by metric: the FE of C_2_H_4_ peaked at 10 wt.% of carbon addition, while *j*_C2H4_ continued to rise until 20 wt.%, reaching 165 mA cm^−2^, approximately 20% higher than the carbon‐free Cu cathode (138 mA cm^−2^). Beyond the optimum, further carbon addition begins to have a negative impact on performance. The mismatched optima for C_2_H_4_ FE and *j*_C2H4_ suggest that increasing the carbon content does not uniformly regulate electrical connectivity, CO_2_ delivery, and accessible Cu‐site density. This indicates that carbon loading meaningfully modulates multiple aspects of the local reaction environment of Cu−carbon cathodes, while loading variation alone cannot identify which structural or interfacial properties of the carbon support govern the observed performance trends.

**FIGURE 1:**
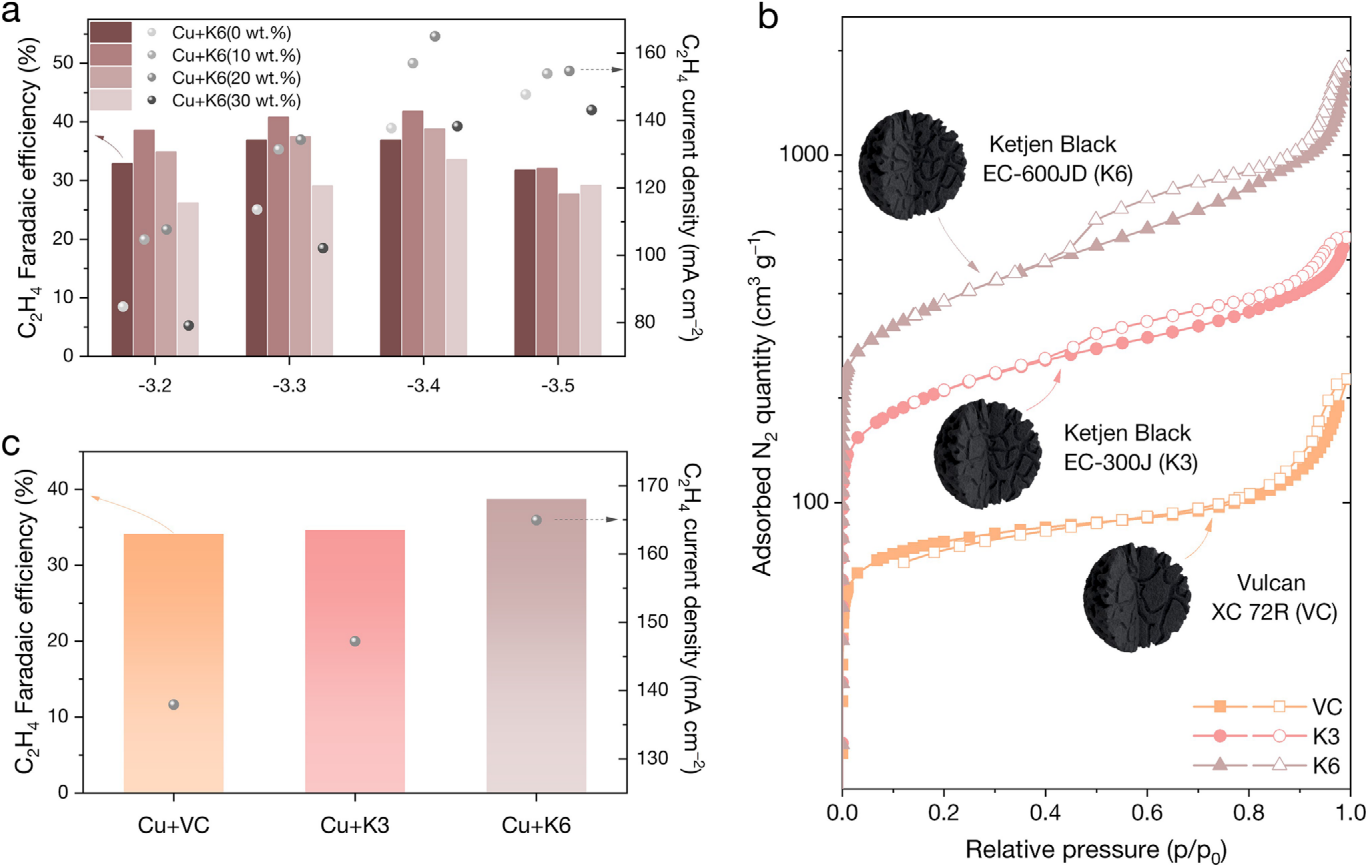
Textural properties of porous carbon black supports and their effect on C_2_H_4_ production. (a) C_2_H_4_ FE and *j*_C2H4_ versus cell voltage (−3.2 to −3.5 V) in an MEA for physically mixed Cu+K6 cathodes with K6 contents of 0, 10, 20, and 30 wt.% relative to the total mass of Cu and carbon. (b) N_2_ adsorption–desorption isotherms at 77 K for VC, K3, and K6; insets schematize their pore networks. (c) Comparison of C_2_H_4_ FE and *j*_C2H4_ at −3.4 V for physically mixed Cu+VC, Cu+K3, and Cu+K6 cathodes at a fixed carbon content of 20 wt.%.

To distinguish carbon‐loading effects from support‐dependent effects, we next held the carbon loading constant and employed commercial carbon blacks with distinct hierarchical textural features under otherwise identical electrode preparation conditions. Specifically, we compared three carbon blacks with hierarchical pore networks: Vulcan XC 72R (VC), Ketjen Black EC‐300J (K3), and Ketjen Black EC‐600JD (K6). N_2_ adsorption–desorption analysis confirmed substantial microporosity (Type I / II isotherms) and mesoporosity (H4‐type hysteresis) in all samples, with the degree of hierarchical structure increasing in the order VC < K3 < K6 (Figure 1b and Table S1). To assess differences beyond pore structure, the three supports were also examined by Raman spectroscopy and C 1s XPS (Figure S2). The Raman spectra showed a modest increase in the I_D_/I_G_ ratio from VC to K3 and K6, while the C 1s XPS spectra were nearly identical in their main peak positions and overall line shapes. These results suggest relatively small differences in the measured carbon surface chemical states and structural disorder, although other support‐dependent interfacial differences cannot be excluded. We then prepared the cathodes by physically mixing Cu with 20 wt.% of each carbon black and compared CO_2_RR performance at −3.4 V to observe the textural effects (Figure 1c). Interestingly, although the C_2_H_4_ FE showed only modest differences across the three carbons, *j*_C2H4_ increased in the order Cu+K6 > Cu+K3 > Cu+VC. This increase in C_2_H_4_ productivity with textural development is consistent with a contribution from the hierarchical pore network to CO_2_ accessibility and transport within the catalyst layer [4, 21].

### 2.2 Preparation and Physicochemical Characterization of Hierarchically Integrated Cu/C Catalysts

With the support‐screening results indicating a transport‐relevant contribution to C_2_H_4_ productivity, we next examined whether spatially integrating Cu nanodomains with the hierarchical carbon framework could more effectively utilize this architecture. We therefore hypothesized that coupling Cu nanodomains with the carbon pore network could shorten effective reactant‐transport paths and support local CO_2_ availability at Cu surfaces [22, 23]. Because K6 exhibited the highest C_2_H_4_ productivity among the physically mixed Cu–carbon electrodes, we first applied the integrated architecture to K6 as a representative highly porous support.

To implement this integrated architecture, we developed an impregnation–reduction route that introduces a Cu precursor throughout the carbon framework and converts it into Cu‐derived nanodomains (Figure 2a). Rigorous washing was applied to suppress external Cu deposition, and scanning electron microscopy (SEM) images predominantly showed aggregates of carbon primary particles without discernible Cu overgrowth (Figure 2b and Figure S3). Aberration‐corrected scanning transmission electron microscopy (STEM) with energy‐dispersive X‐ray spectroscopy (EDS) mapping showed that the Cu nanodomains were distributed throughout the carbon framework (Figure 2c and Figure S4). The Cu particle size ranged from ∼5 to 20 nm, with the nanodomains distributed throughout the carbon aggregates and interparticle regions, consistent with the integrated Cu–carbon architecture. High‐resolution transmission electron microscopy (HR‐TEM) frequently revealed lattice fringes consistent with Cu_2_O (200), while metallic Cu fringes were also observed (Figure 2d and Figures S5 and S6), consistent with mixed Cu^0^/Cu^+^ nanodomains.

**FIGURE 2:**
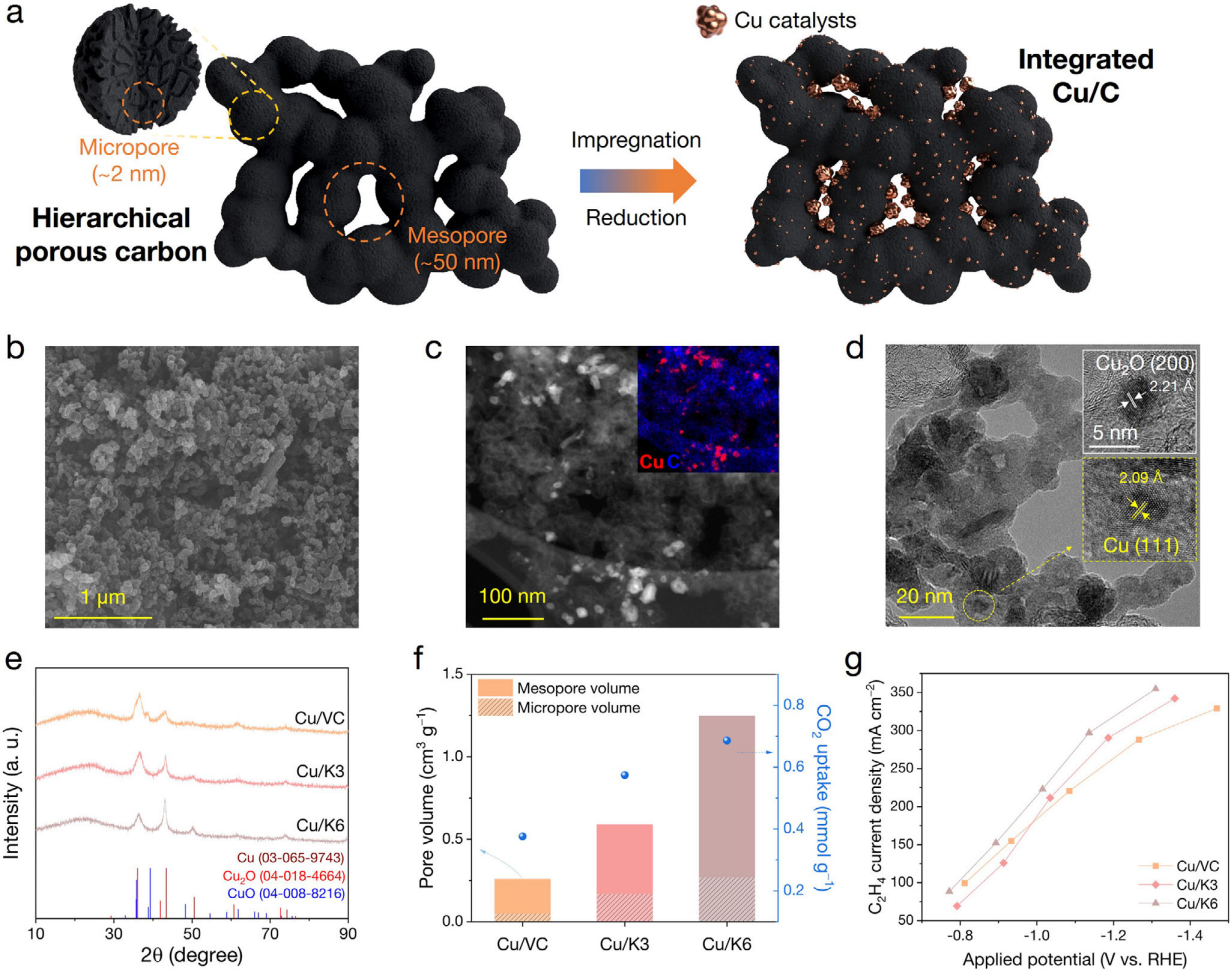
Hierarchically integrated Cu/C catalysts: synthesis, characterization, and CO_2_RR performance. (a) Schematic comparison of the hierarchical porous carbon framework before and after Cu integration, illustrating Cu nanodomains distributed throughout the resulting Cu/C architecture. (b) SEM image showing aggregates of the primary carbon particles without discernible Cu overgrowth. (c) STEM image showing Cu nanodomains distributed throughout the carbon framework; accompanying EDS map shows Cu (red) and C (blue). (d) TEM image of Cu nanodomains associated with the carbon framework; inset: lattice fringes indexed to Cu_2_O (200) and Cu(111). (e) XRD patterns of Cu/C samples. (f) Micropore and mesopore volumes of Cu/C samples and the corresponding CO_2_ uptakes. (g) *j*_C2H4_ as a function of iR‐corrected cathode potential during CO_2_RR for Cu/C samples in a flow‐cell.

After validating the integrated architecture using K6, we extended the same impregnation–reduction protocol to VC and K3, yielding Cu/VC and Cu/K3. Morphological analyses indicated that Cu‐derived nanodomains generally formed throughout the carbon frameworks; however, for VC, which has less‐developed textural features, a larger fraction of Cu species appeared on or around the external surfaces of carbon aggregates (Figures S3–S6). X‐ray diffraction (XRD) detected metallic Cu together with Cu_2_O in all samples (Figure 2e). Notably, Cu/K6 exhibited the most intense Cu(111) reflections, consistent with a greater contribution from crystalline metallic Cu domains. Consistent with this trend, ex situ X‐ray absorption spectroscopy (XAS) showed that the average Cu oxidation state generally decreased as the textural development of the carbon support increased, with Cu/K6 being the most reduced (Figures S7–S10 and Supporting Note). Because Cu catalysts can undergo structural and oxidation‐state changes under cathodic CO_2_RR conditions, we examined whether the differences observed ex situ persisted during operation [24, 25]. Operando Cu K‐edge extended X‐ray absorption fine structure (EXAFS) showed that all three Cu/C catalysts evolved toward predominantly metallic‐like coordination, with a clear Cu–Cu contribution emerging under cathodic polarization and remaining dominant as the current density increased (Figure S11). These observations indicate that the initial differences in average Cu coordination became less pronounced during CO_2_RR, highlighting the need to consider support‐dependent factors beyond the initial Cu oxidation state when interpreting the catalytic behavior.

N_2_ adsorption–desorption at 77 K indicated that the catalysts largely retained the hierarchical porosity of their parent carbons, with some pore blocking but the same overall order in total pore volume (Cu/K6 > Cu/K3 > Cu/VC; Figure 2f, Figure S12 and Table S2). To probe whether this porous support architecture promotes CO_2_ enrichment within the Cu/C catalysts, we measured CO_2_ adsorption isotherms (Figure S13). CO_2_ uptake increased with the extent of accessible microporosity, consistent with the higher CO_2_ affinity of narrow pores, suggesting that Cu/K6 provides a greater capacity for CO_2_ enrichment within the carbon pore network. Although adsorption alone does not demonstrate enhanced transport under operating conditions, these data establish a porosity‐dependent CO_2_ enrichment capacity that motivates the electrochemical tests that follow.

To provide a cleaner basis for comparing carbon‐support architectures in integrated Cu/C catalysts, CO_2_RR performance was evaluated in a three‐electrode flow‐cell under galvanostatic operation, allowing direct monitoring of the cathode potential and separation of cathode behavior from the anode‐ and membrane‐related contributions (Figure 2g and Figure S1). At identical total current densities, cathodes based on supports with more developed hierarchical porosity operated at lower cathodic overpotentials, an advantage that became more pronounced at higher current densities. Accordingly, *j*_C2H4_ increased with carbon‐support porosity at high current density, with the Cu/K6 catalyst reaching a *j*_C2H4_ of 355 mA cm^−2^ and a C_2_H_4_ FE of 50.7% (Figure S14). This behavior is consistent with enhanced local CO_2_ availability in the Cu catalyst microenvironment associated with increasing porosity, which can mitigate reactant depletion and mass‐transport limitations [4, 26]. Across the series, the maximum *j*_C2H4_ showed an apparent positive association with CO_2_ uptake (Figure S15) and stronger apparent correlations with Brunauer−Emmett−Teller (BET) surface area and micropore volume, suggesting that microporous channels may provide effective pathways for CO_2_ enrichment and transport near Cu sites, thereby promoting higher C_2_H_4_ productivity under high‐rate conditions [15, 27]. Notably, the C_2_H_4_ FE varied only modestly across Cu/VC, Cu/K3, and Cu/K6, whereas the cathode potential and resulting *j*_C2H4_ changed more substantially. Together with the predominantly metallic‐like Cu coordination observed across the catalysts during operation, this pattern suggests that the support‐dependent pore architecture influences how effectively CO_2_ is supplied to Cu nanodomains integrated with the carbon framework under transport‐influenced conditions.

### 2.3 Architecture‐Dependent CO_2_RR Performance of Cu–K6 Cathodes

Although carbon porosity is linked to enhanced C_2_H_4_ productivity through improved CO_2_ transport, distinguishing the contribution of pore structure from that of Cu–carbon integration within the carbon framework requires further examination. To this end, two Cu–K6 cathodes sharing the same K6 support but differing in their integration architecture were prepared: a physically mixed benchmark comprising commercial Cu nanoparticles (∼25 nm) and K6, denoted Cu+K6, and the impregnation‐derived, hierarchically integrated Cu/K6 catalyst. An ideal control would preserve the Cu morphology and composition while changing only its integration with the carbon framework. However, Cu isolated from Cu/K6 did not retain the same particle size or oxidation state and exhibited markedly inferior CO_2_RR performance, making it unsuitable as a matched control. Commercial Cu nanoparticles were therefore selected as a reproducible practical benchmark for physical mixing with the same K6 support. Cu+K6 contained 20 wt.% Cu, comparable to the 22.2 wt.% Cu content of Cu/K6 determined by inductively coupled plasma mass spectrometry (ICP‐MS) analysis. Accordingly, this comparison provides a reproducible architecture‐level benchmark between physically mixed and hierarchically integrated Cu–K6 cathodes using the same carbon support and comparable Cu contents, rather than a strictly size‐ and structure‐matched single‐variable control.

The contrasting Cu–carbon architectures are schematically illustrated in Figure 3a,b. Cross‐sectional secondary‐electron and backscattered‐electron images further revealed relatively coarse and discrete Cu‐rich domains in the physically mixed Cu+K6 cathode, whereas smaller Cu‐containing domains were distributed throughout the catalyst layer of Cu/K6 (Figure 3c,d). Semiquantitative EDS analysis of the displayed cross‐sectional regions showed comparable local Cu fractions of approximately 10% for Cu+K6 and 12% for Cu/K6, consistent with the comparable Cu contents of the two cathodes. Operando Cu K‐edge EXAFS was subsequently used to assess whether differences in the initial Cu coordination environments persisted under CO_2_RR conditions (Figure 3e). Cu+K6 and Cu/K6 exhibited distinct coordination features at open‐circuit voltage (OCV); however, following cathodic pre‐reduction and operation at increasing current densities, both catalysts evolved toward predominantly metallic‐like Cu coordination, with their spectra approaching that of a Cu foil reference. Although Cu structural characteristics can influence C─C coupling and C_2+_ formation [28], the observed convergence in Cu coordination indicates that the initial differences in Cu state become less pronounced during operation and are unlikely to be the dominant origin of the performance difference between the two cathodes.

**FIGURE 3:**
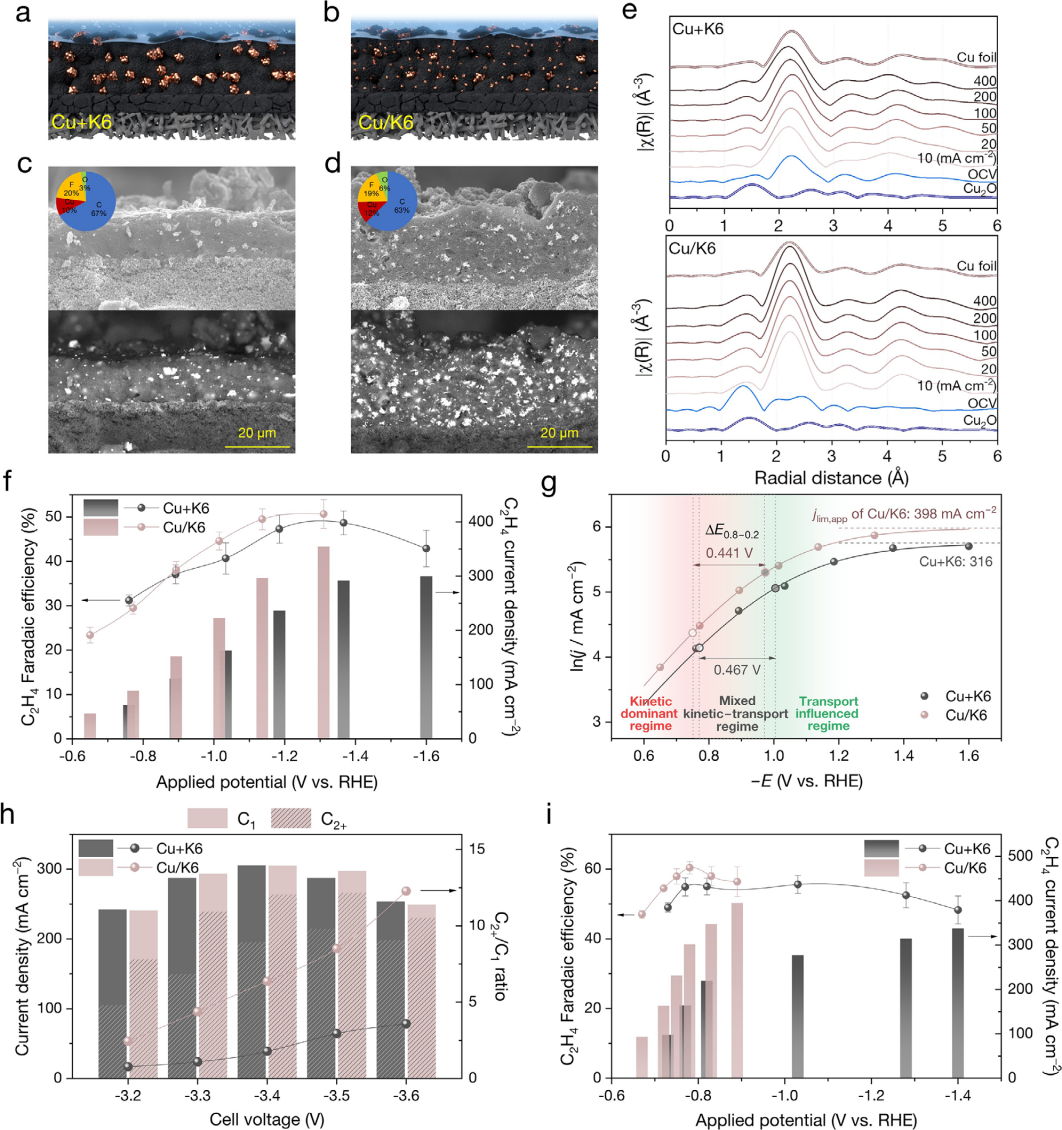
Structural and electrochemical comparison of physically mixed Cu+K6 and hierarchically integrated Cu/K6 cathodes. (a, b) Schematic illustrations of the catalyst‐layer architectures of (a) physically mixed Cu+K6 and (b) hierarchically integrated Cu/K6. (c, d) Cross‐sectional secondary‐electron and backscattered‐electron SEM images of (c) Cu+K6 and (d) Cu/K6; the inset shows the semiquantitative elemental compositions in wt.% determined by EDS. (e) Operando Cu K‐edge EXAFS spectra of Cu+K6 and Cu/K6 collected at OCV, following cathodic pre‐reduction and during CO_2_RR operation at increasing current densities; Cu foil and Cu_2_O spectra are included as references. (f) C_2_H_4_ Faradaic efficiency and *j*_C2H4_ as a function of the cathode potential in flow‐cell CO_2_RR. (g) Mixed kinetic–transport analysis of *j*_C2H4_ plotted versus *−E* using data obtained during CO_2_RR in a flow‐cell, separating kinetic, mixed‐control, and transport‐influenced regimes. (h) Partial current densities of C_1_ and C_2+_ products at cell voltages from −3.2 to −3.6 V in an MEA, with corresponding C_2+_/C_1_ ratios. (i) C_2_H_4_ Faradaic efficiency and *j*_C2H4_ as a function of the cathode potential during CORR in a flow‐cell.

Following these structural and operando comparisons, under galvanostatic operation in a flow‐cell, Cu/K6 exhibited markedly improved performance compared with Cu+K6 (Figure 3f and Figure S16). At identical total current densities, Cu/K6 operated at substantially less negative cathode potentials, with a difference of ∼0.3 V at 700 mA cm^−2^, while simultaneously delivering ∼20% higher *j*_C2H4_. To examine how this performance difference developed across the polarization range, we fitted the *j*–*E* polarization data using a Koutecký–Levich–type mixed‐control expression (Figure 3g) [29]. The fitted parameters are used here as comparative descriptors of the macroscopic polarization behavior within the coupled kinetic–transport framework, rather than as independently resolved intrinsic kinetic or mass‐transfer constants. Fitting yielded a higher apparent limiting current density for Cu/K6 (*j*_*lim*,*app*_ = 398 mA cm^−2^) than for Cu+K6 (316 mA cm^−2^), indicating a higher apparent current ceiling under transport‐influenced conditions (Table S3). In contrast, the apparent kinetic parameters were comparable for the two cathodes (Cu/K6: *j*_0,*app*_ = 0.888 mA cm^−2^, *k_app_* = 6.29 V^−1^; Cu+K6: *j*_0,*app*_ = 0.817 mA cm^−2^, *k_app_* = 5.93 V^−1^). The potential gap between the two electrodes was small near 20% of *j*_*lim*,*app*_ (∼20 mV) but widened near 80% of *j*_*lim*,*app*_ (∼45 mV). Within this fitting framework, the distinction between the two cathodes therefore became more pronounced as their current approached the apparent limiting values. This behavior is consistent with hierarchical Cu–carbon interaction helping sustain CO_2_ supply to Cu nanodomains under high‐rate conditions, thereby mitigating reactant‐transport limitations relative to the physically mixed Cu+K6 cathode [30, 31]. In contrast to C_2_H_4_, the partial current densities for the competing hydrogen evolution reaction (HER) showed little curvature and were well described by a near‐linear trend over the measured window, indicating a different polarization behavior from CO_2_RR (Figure S17).

This performance advantage was also maintained under device‐relevant conditions in a zero‐gap MEA configuration, where CO_2_RR product distributions differed markedly between the two cathodes (Figure 3h and Figure S18). At low cell potentials, Cu+K6 produced a higher fraction of C_1_ products, whereas Cu/K6 already favored C_2+_ products; this disparity became increasingly pronounced at more negative potentials, reaching C_2+_/C_1_ ratios of 3.6 for Cu+K6 and 12.3 for Cu/K6 at −3.6 V. This enhanced selectivity contrast in the MEA is consistent with the gas‐rich, water‐lean environment, in which the hierarchically integrated Cu–K6 architecture may limit electrolyte intrusion and maintain continuous CO_2_ transport pathways. Such a microenvironment may favor the local availability of CO‐derived intermediates, while differences in water transport and OH^−^ accumulation may concurrently alter the local interfacial pH [32]; both effects can provide conditions favorable for C–C coupling [21, 33, 34]. The stronger effect on C_2+_ selectivity may therefore arise from the coupled influence of CO‐derived intermediate availability and local interfacial conditions that favor C─C coupling.

To better understand the origin of the performance advantage of Cu/K6 in the MEA configuration, we further examined the possible contribution of electrochemically accessible interfacial area and preparation history. The double‐layer capacitance (C_dl_) was 5.3 mF cm^−2^ for Cu+K6 and 6.1 mF cm^−2^ for Cu/K6 (Figure S19). After normalization by these values, Cu/K6 retained a higher C_dl_‐normalized *j*_C2H4_ than Cu+K6 at −3.4 V (33.5 vs. 30.2 mA mF^−1^). However, the K6‐only electrode exhibited an even higher C_dl_ of 7.3 mF cm^−^^2^, indicating that the measured capacitance includes a substantial contribution from the carbon support and does not exclusively reflect the accessible Cu surface area. We next examined whether preparation procedure affected Cu properties and CO_2_RR performance. As a control, commercial Cu nanoparticles were subjected to the same treatment sequence used for Cu/K6 synthesis, yielding treated Cu (t‐Cu). XRD showed little change in the crystalline Cu phase after treatment, whereas TEM indicated increased particle agglomeration (Figure S20). When physically mixed with K6, t‐Cu+K6 exhibited lower CO_2_RR performance than the Cu+K6 cathode under otherwise identical MEA conditions. Its C_dl_ (4.8 mF cm^−2^) was also lower than Cu+K6, consistent with reduced electrochemically accessible interfacial area. Thus, the preparation treatment can influence Cu properties and interfacial accessibility, but does not fully account for the performance difference between the physically mixed and integrated cathodes. Together with the C_dl_‐normalized comparison, these results remain consistent with an architecture‐dependent contribution from hierarchical Cu–carbon integration.

The operational behavior of Cu/K6 and Cu+K6 was further evaluated under extended CO_2_RR operation at −3.3 V in the MEA configuration (Figure S21). Although the C_2_H_4_ selectivity of Cu/K6 gradually decreased during the initial period, it subsequently reached a relatively stable regime under continuous operation. Post‐reaction SEM revealed pronounced crystalline deposits across the electrode surface, while STEM‐EDS mapping showed substantial accumulation of K‐containing species within the catalyst layer (Figure S22). Cu remained broadly distributed throughout the carbon framework, although localized Cu agglomeration was also observed after operation. These observations suggest that salt accumulation, together with localized Cu agglomeration, may have contributed to the gradual performance decay by perturbing pore accessibility, local wetting, and reactant transport. Despite this degradation, Cu/K6 maintained a higher C_2_H_4_ FE than Cu+K6 throughout the shared testing period and continued operating after the Cu+K6 was terminated at ∼25 h due to unstable cell operation, demonstrating a comparative operational advantage under the tested MEA conditions.

To further examine whether the integrated architecture also influences CO‐derived intermediate availability beyond initial CO_2_ activation, CO reduction reaction (CORR) experiments were performed in the same flow‐cell setup (Figure 3i and Figure S23). By using CO as the reactant, CORR bypasses CO_2_ dissolution, electrolyte acid−base speciation, and the initial CO_2_‐to‐*CO conversion step, thereby placing greater emphasis on CO‐derived reactant availability at the cathode [35]. CORR therefore provides a complementary means of assessing how carbon architecture and Cu–carbon integration influence reactant supply at the cathode. Under CORR conditions, a substantially larger potential difference emerged between the two cathodes than in CO_2_RR: at a total current density of 700 mA cm^−2^_,_ Cu/K6 operated at a cathode potential ∼0.5 V less negative than Cu+K6. This pronounced separation is consistent with more favorable CO supply to Cu sites in the integrated architecture under high‐rate conditions. Cu/K6 also achieved a higher *j*_C2H4_ of 394 mA cm^−2^ compared with 338 mA cm^−2^ for Cu+K6. These CORR results support the interpretation that the integrated architecture provides more favorable CO‐derived reactant availability under high‐rate conditions, complementing the trends observed in both flow‐cell CO_2_RR and MEA measurements [21, 33, 36].

### 2.4 Multiphysics Simulations of CO_2_ Transport in Integrated Cu–Carbon Architectures

To examine the correlation between structural architecture and electrochemical performance, a two‐dimensional multiphysics model was developed (Figure 4). The computational domain represents a unit of the cathode interface, comprising a gas flow channel, a gas diffusion layer, a catalyst layer, and an electrolyte channel. The geometry distinguishes the hierarchically integrated Cu/K6 architecture from the physically mixed Cu+K6 architecture, with characteristic Cu particle sizes and spatial distributions derived from SEM and STEM‐EDS analyses (Figure 3a–d). A representative single‐pore model was employed to achieve a balance between nanoscale resolution and computational tractability. The model resolves coupled transport and reaction kinetics for CO_2_RR with HER as a competing reaction under the experimental conditions (1 M KOH and dry CO_2_ feed). The same set of electrochemical kinetic parameters (Table S5) was applied to both architectures. Rather than treating mass transfer coefficients as fixed intrinsic parameters, transport was modeled as a system‐dependent dynamic parameter governed by local hydrodynamics and geometric configurations [37]. Transport parameters were therefore tailored to our nanostructured flow‐cell environment to represent liquid‐phase transport within the pore architecture and reflect reactant availability at Cu nanodomains integrated within the carbon framework [38]. The simulations qualitatively reproduced the experimentally observed performance trends of the key products C_2_H_4_, CO, and H_2_ (Figure 4c,d, and Figure S24). Because both models shared the same kinetic parameters, the simulated difference between them does not arise from differences in the assumed kinetics. While both catalysts show increasing *j*_C2H4_ at low overpotentials, Cu+K6 enters a transport‐limited regime at high current densities, consistent with a threshold imposed by mass‐transfer limitations. In contrast, Cu/K6 sustains elevated *j*_C2H4_ under the same conditions, highlighting the structural advantage of hierarchically integrating Cu nanodomains with the carbon pore network. These trends provide a basis for visualizing how the integrated architecture mitigates reactant depletion within the catalyst microenvironment under demanding high‐rate operating conditions.

**FIGURE 4:**
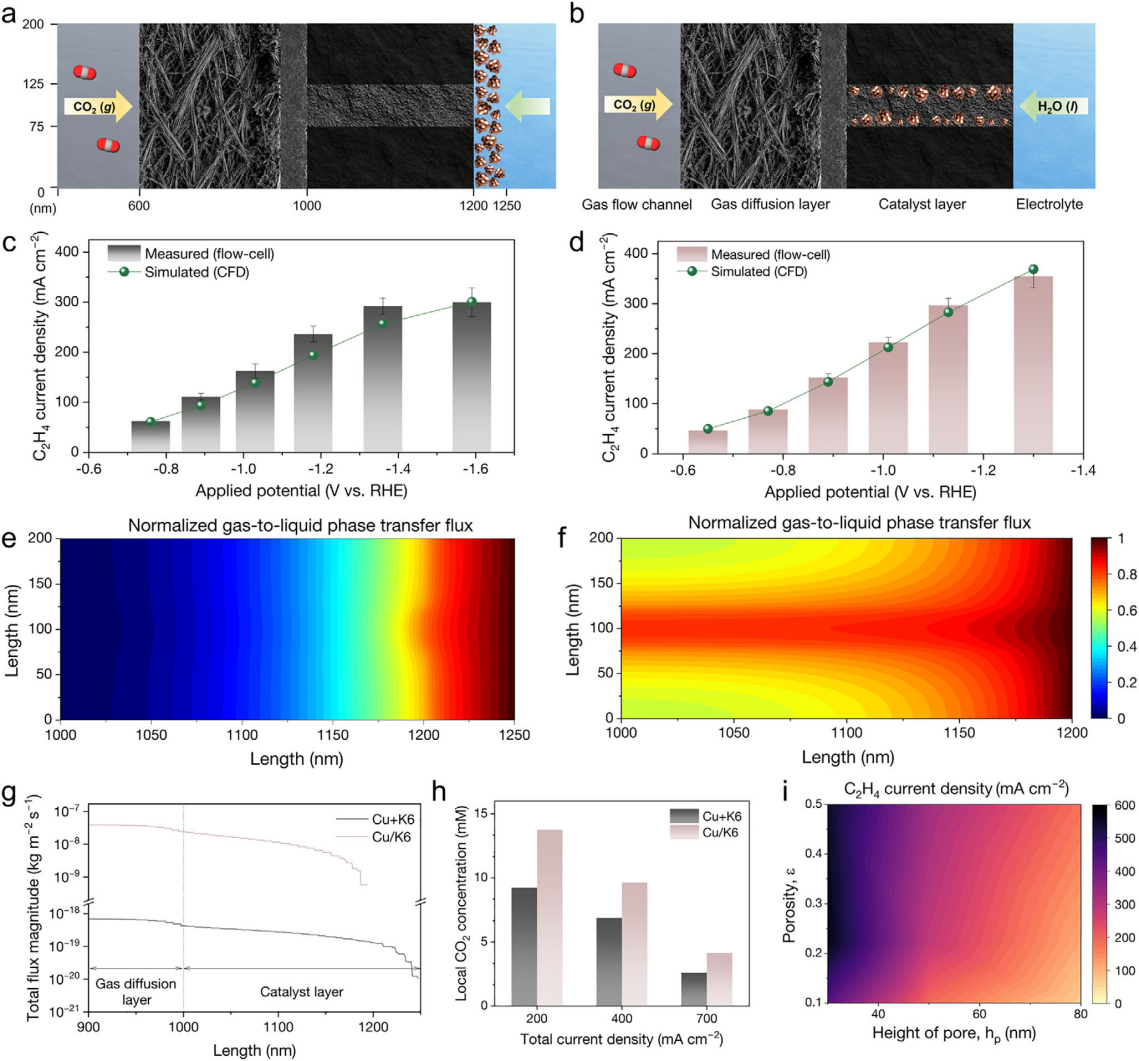
Multiphysics simulation domains and CO_2_ transport and microenvironment in Cu+K6 and Cu/K6 catalyst layers. (a, b) Two‐dimensional computational domains comprising the gas flow channel, gas diffusion layer, catalyst layer, and electrolyte flow channel for (a) the physically mixed Cu+K6 architecture and (b) the hierarchically integrated Cu/K6 architecture. (c, d) Comparison of experimentally measured flow‐cell and simulated local *j*_C2H4_ for (c) Cu+K6 and (d) Cu/K6. (e, f) Contour maps of the normalized gas‐to‐liquid CO_2_ phase‐transfer flux within the catalyst‐layer domain for (e) Cu+K6 and (f) Cu/K6. (g) One‐dimensional profiles of the magnitude of the total CO_2_ flux across the gas diffusion and catalyst layer. (h) Volume‐averaged residual dissolved CO_2_ concentration within the catalyst layer as a function of total current density. (i) Sensitivity analysis of simulated *j*_C2H4_ as a function of pore height and porosity in the single‐pore model.

We next examined the local transport environment of both gaseous and dissolved CO_2_ within the catalyst layer to examine the transport‐related basis of the performance disparity between Cu/K6 and Cu+K6. Based on the Maxwell‐Stefan model, the diffusive flux of CO_2_ is strongly influenced by the transport path length [39]. Accordingly, within the model, hierarchical integration of Cu with the carbon pore network in Cu/K6 reduces the effective diffusion distance between the gas‐liquid interface and the active sites relative to the physically mixed control (Cu+K6). At a total current density of 600 mA cm^−2^, the simulated contour maps show a clear difference in CO_2_ transport behavior and phase‐transfer flux between the two architectures (Figure 4e–g and Figures S25–S27). In the Cu/K6 model, the integrated geometry maintains CO_2_(g) access to the reactive regions and supports a stronger gas‐to‐liquid transfer flux. As CO_2_(aq) is consumed near active sites, a local depletion develops that increases the driving force for dissolution, thereby sustaining an inward flux that replenishes the consumed reactant [40]. In contrast, the Cu+K6 model exhibits a weaker phase‐transfer flux and lower net CO_2_ supply to the catalyst, consistent with more severe local reactant depletion under high‐rate operation. We also evaluated the local water distribution as another key microenvironment descriptor. Taken together, the simulations support the interpretation that spatial differences in CO_2_ availability and the associated transport and phase‐transfer limitations contribute to the performance divergence between the two architectures [41].

Beyond shortened diffusion paths, the integrated architecture of Cu/K6 is predicted to increase the density of effective triple‐phase boundaries and retain CO_2_ within the pore network [42]. In the hierarchical model, this architecture provides a high surface‐to‐volume ratio and places Cu sites in immediate proximity to gas‐filled macropores, enabling localized CO_2_ flux that sustains reactant availability by reducing reliance on long‐range diffusion from the bulk electrolyte. Because the model calculates the liquid content from water activity rather than from wettability, this prediction assumes similar wetting of the two catalyst layers (Figure S28). Simultaneously, the confined pore geometry limits convective loss of dissolved CO_2_ to the bulk electrolyte and prolongs its residence within the pore channels. In Cu+K6, dissolved CO_2_ is more readily transported away by the electrolyte flow before reaching Cu sites, whereas the Cu/K6 architecture promotes the retention of CO_2_(aq) within its narrow channels (Figure 4h). Together, these modeled effects mitigate local reactant depletion under high current densities and maintain a transport‐favorable microenvironment that promotes C─C coupling [33, 34, 43].

To assess how the structural descriptors of the catalyst layer influence C_2_H_4_ productivity, we performed a sensitivity analysis at 600 mA cm^−2^ by varying the pore height (h_p_), which corresponds to the pore diameter in the single‐pore model, and porosity (ε) in the single‐pore model. As shown in the contour map (Figure 4i and Figure S29), *j*_C2H4_ exhibits a strong dependence on both parameters. Increasing h_p_ leads to a monotonic decrease in *j*_C2H4_, consistent with a longer diffusion path and a greater CO_2_ depletion toward deeper regions of the pore. Conversely, increasing porosity (ε) generally enhances *j*_C2H4_ by increasing the effective diffusivity. Increasing the porosity from 0.1 to 0.5 enhances the effective diffusivity by approximately 11.2‐fold. This substantial improvement in transport capacity allows the system to sustain a higher catalyst utilization rate, thereby increasing the peak *j*_C2H4_ from 188.3 to 328.4 mA cm^−2^. The simulated trend provides a mechanistic rationale consistent with the VC–K3–K6 screening results: carbons with more developed textural features and greater accessible pore connectivity can better mitigate CO_2_ transport losses and thereby sustain higher C_2_H_4_ productivity under high‐rate operation.

The present model has several simplifications. The single‐pore geometry does not resolve the broad pore‐size distribution, the heterogeneous pore connectivity, salt precipitation, or non‐uniform wetting of the real catalyst layer. The catalyst layer in the model contains both gas and liquid phases. The liquid content is calculated from the local water activity, and gaseous CO_2_ diffuses into the pores to form the triple‐phase boundary. The model does not compute how wettability and capillary pressure distribute the liquid within the pores, and it does not capture flooding. The simulated local CO_2_ profiles were not verified by spatially resolved measurements. Within the single‐pore geometry, increasing the pore depth from 200 to 300 nm raised the volume‐averaged CO_2_ concentration from 5.23 to 5.83 mol m^−3^ (Figure S30). This depth runs from the gas side toward the electrolyte and is perpendicular to the pore height (h_p_) in Figure 4i. Because CO_2_ enters the pore from the side opposite to the pore opening, a deeper pore shortens the gas‐phase diffusion length and raises the CO_2_ concentration. We further tested whether the kinetic parameters calibrated with the flow‐cell data remain applicable to the MEA. In the MEA, water and ion transport are coupled with membrane hydration. Water also serves as the proton source for the hydrogenation steps of CO_2_ reduction [44] and for the HER [45]. The kinetic parameters were kept unchanged. The gas‐side boundary was set to water vapor saturated at 30°C, and the effective CO_2_ diffusivity was adjusted to match only the measured total current density. Under these conditions, the simulated *j*_C2H4_ agreed with the measured MEA values within 10.6% for Cu/K6 and 6.2% for Cu+K6 (Figure S31). This agreement suggests that the kinetic parameters for C_2_H_4_ formation lie in a physically reasonable range beyond the calibration condition. In contrast, the model underpredicted the *j*_H2_ (Figure S32) and overpredicted the *j*_CO_. The simulated water activity in the catalyst layer stayed between 0.9111 and 0.9120 (Figure S33). The model therefore does not capture local variations in water availability. The *j*_H2_ and *j*_CO_ are likely affected by MEA‐specific factors that the model does not resolve, such as the local water distribution, independent electrode potentials, the local pH, and the local cation concentration. These factors may also contribute to the larger C_2+_/C_1_ ratio enhancement observed in the MEA. Taken together, the MEA calculations reproduced the *j*_C2H4_ reasonably well using the unchanged kinetic parameters after adjustment of the effective CO_2_ diffusivity, but did not reproduce the *j*_H2_ and *j*_CO_. Despite these limitations, the model remains useful for comparing the relative CO_2_ transport behavior of the physically mixed and hierarchically integrated architectures, providing mechanistic insight into the role of Cu–carbon integration without fully reconstructing the multiphase environment of the catalyst layer.

## 3 Conclusions

In this study, we elucidated how incorporating porous carbon into Cu‐catalyzed CO_2_RR electrodes benefits C_2_H_4_ electrosynthesis, with an emphasis on pore‐derived effects. We observed a clear structure–performance relationship across porous carbon supports and associated this trend primarily with pore‐mediated transport that supports local CO_2_ and CO‐derived intermediate availability. Guided by this insight, we developed hierarchically integrated Cu/C catalysts in which Cu nanodomains were distributed throughout the carbon framework. These catalysts delivered higher *j*_C2H4_ as the hierarchical pore architectures became more developed. By comparing physically mixed and hierarchically integrated Cu–K6 cathodes, we found that the integrated architecture shifted the apparent transport‐limitation threshold to higher current densities, enabling lower overpotentials while increasing *j*_C2H4_ and selectivity at the same total current densities. CFD simulations provided a mechanistic rationale consistent with these trends, indicating that the integrated architecture sustains reactant availability by shortening effective diffusion paths and mitigating local CO_2_ depletion. Collectively, these findings establish a rational design principle for porous‐carbon‐based Cu CO_2_RR cathodes: hierarchical pore networks can serve as effective CO_2_ transport pathways, and their benefit can be maximized by spatially integrating Cu nanodomains with these transport pathways.

## 4 Experimental Section

### 4.1 Materials

Copper(II) nitrate trihydrate, sodium borohydride, Cu nanoparticles (CuNPs, 25 nm), polytetrafluoroethylene (PTFE) dispersion (60 wt.% in H_2_O), potassium bicarbonate, potassium hydroxide, dimethyl sulfoxide (DMSO), phenol, deuterium oxide (D_2_O), and D_2_O containing tetramethylsilane (0.05 wt.%) were purchased from Sigma‒Aldrich. Commercial Ni foam was supplied by MTI Korea. Iridium(IV) oxide (IrO_2_) was obtained from Thermo Fisher Scientific, and isopropyl alcohol (IPA) was purchased from Daejung Chemicals & Metals. Commercial carbon blacks: Vulcan XC 72R (VC), Ketjen Black EC‐300J (K3), and Ketjen Black EC‐600JD (K6) were supplied by Infochems. Nafion solution (D520, 5 wt.%) was purchased from Omniscience. Anion exchange membranes (Fumasep FAA‐3‐PK‐130 and PiperION, 15 µm), carbon paper (Sigracet 39BB), and platinized titanium screen (0.002 in.) were obtained from Fuel Cell Store. High‐purity CO_2_, CO, H_2_, and Ar gases were purchased from Shinyang Medicine. Deionized water (DIW) with a resistivity of 18.2 MΩ·cm was used to prepare all aqueous solutions.

### 4.2 Catalyst Preparation

Commercial carbon blacks were used as hierarchical porous carbon supports and sieved to a particle size below 63 µm. Copper(II) nitrate trihydrate (3.02 g) was dissolved in 25 mL of IPA, and 100 mg of carbon supports were dispersed in 50 mL of IPA. Each dispersion was sonicated separately for 1 h, then combined in a single vessel and homogenized via ultrasonic impregnation for 3 h. The copper precursor‐impregnated carbon supports were collected by vacuum filtration and gently washed stepwise with 150 mL of IPA to remove the residual precursor from the external surface. The collected materials were vacuum‐dried at room temperature for 4 h and subsequently washed once with DIW. The dried powder was then vigorously mixed with 20 mL of DIW using a vortex mixer, vacuum‐filtered, and further washed with 150 mL of DIW to remove residual surface precursor. Finally, the materials were vacuum‐dried overnight at room temperature. The resulting copper precursor‐impregnated carbon supports were denoted as Cu^x+^/C.

Sodium borohydride, serving as a reducing agent, was dispersed in 75 mL of IPA under vigorous stirring and heated to 75°C for 30 min. The Cu^x+^/C powder was then added to the solution and sonicated for 30 min. The resulting dispersion was further heated at 75°C for 3 h under continuous stirring. The Cu‐integrated carbon supports were collected by vacuum filtration, followed by IPA washing, vacuum drying, and DIW washing, using the same procedure as for Cu^x+^/C. After the final DIW wash, the materials were vacuum‐dried overnight at 60°C. The resulting materials were denoted as Cu/C. The Cu/C catalysts were designated as Cu/VC, Cu/K3, and Cu/K6, depending on the type of carbon support used. Treated commercial Cu (t‐Cu) was prepared by subjecting commercial Cu nanoparticles to the same solvent exposure, NaBH_4_ reduction, washing, and drying procedures used for the preparation of Cu/K6.

### 4.3 Electrode Preparation

To fabricate the Cu/C cathode for membrane electrode assembly (MEA), 10 mg of Cu/C catalyst and 120 µL of diluted PTFE dispersion (5 wt.% in H_2_O) were dispersed in 2 mL of IPA and ultrasonicated for 30 min. The resulting Cu/C ink was spray‐coated onto a 2.5 × 2.5 cm^2^ piece of Sigracet 39BB carbon paper while heating the substrate to 70°C, followed by vacuum‐drying at room temperature for 2 h. For the preparation of physically mixed Cu+C MEA cathodes, 20 mg of commercial CuNPs, 80 mg of carbon support, and 1.2 mL of diluted PTFE dispersion were dispersed in 20 mL of IPA and ultrasonicated for 30 min. The Cu‐to‐carbon mass ratio of 1:4 was selected to approximate the Cu content of Cu/K6 determined by ICP‐MS analysis (22.2 wt.%). A total of 2 mL of the resulting Cu+C ink was spray‐coated onto carbon paper using the same procedure. The t‐Cu+K6 cathode was prepared using the same procedure by replacing commercial CuNPs with t‐Cu.

For flow‐cell cathodes, the amount of catalyst ink was doubled, and the PTFE dispersion was replaced with an equal volume of Nafion binder (5 wt.%). The ink was then spray‐coated onto 3.5 × 3.5 cm^2^ carbon paper. A cathode window of 2.5 × 2.5 cm^2^ was used in the flow‐cell configuration, resulting in the same effective electrode area as in the MEA configuration.

Iridium(IV) oxide supported on platinized titanium mesh (IrO_2_/Pt‒Ti mesh) was used as the anode in the MEA configuration. To prepare the anode, 300 mg of IrO_2_ and 300 µL of 5 wt.% Nafion solution was dispersed in 10 mL of IPA and ultrasonicated for 1 h. A total of 1 mL of the resulting IrO_2_ ink was evenly spray‐coated onto four 2.5 × 2.5 cm^2^ pieces of platinized titanium mesh. All four IrO_2_/Pt‒Ti mesh pieces were used for each experimental run. Commercial Ni foam was used as the anode in the flow‐cell configuration and was cut to a size of 2.5 × 2.5 cm^2^.

### 4.4 Electrochemical Experiments

Electrochemical experiments were conducted using an electrochemical workstation (SP‐150e potentiostat, BioLogic) equipped with a 20 A current booster (VMP‐3B‐20, BioLogic) and a 5 cm^2^ electrolyzer (SKU: 68732, Dioxide Materials). The catholyte cell for the flow‐cell system was fabricated from PTFE with a window size of 2.5 × 2.5 cm^2^ and a thickness of 5 mm. All gaskets used in both systems were fabricated to match the thickness of the corresponding cathode or anode. Electrolytes were continuously circulated using a peristaltic pump (EMS‐Tech). In the MEA system, humidified CO_2_ (100% relative humidity at 30°C) was introduced as the feed gas to prevent membrane dehydration.

Gas products were analyzed using gas chromatography (GC; 8890 B, Agilent Technologies) equipped with a flame ionization detector and a thermal conductivity detector. A ShinCarbon column was employed with Ar as the carrier gas. Liquid products were quantified by ^1^H nuclear magnetic resonance (^1^H NMR; Avance III HD, Bruker) spectroscopy with water suppression, using DMSO and phenol as internal standards. Liquid samples were collected from the anolyte in the MEA system and from the catholyte in the flow‐cell setup. The Faradaic efficiency (FE) of each product was calculated using the following equation:

(1) $$FE_{\mathrm{product}}\;\left(\%\right)=\frac{i_{\mathrm{product}}}{i_{\mathrm{total}}}\;\times 100=\frac{n_{\mathrm{product}}\;\times \;F\;\times \;N}{i_{\mathrm{total}}}\;\times 100$$

where *i_product_* is the partial current corresponding to the product, *i_total_* is the total current, *n_product_* is the molar production rate of the product determined by GC or ^1^H NMR, F is the Faraday constant (96 485 C mol^‒1^), and N is the number of electrons transferred per molecule of product formed from CO_2_ or CO. The product distribution is presented as the average of at least three independent measurements, with the corresponding error bars. All cathodic current densities are reported as positive values.

To report cathode potentials on a consistent scale in the flow‐cell, the measured potentials were converted from Ag/AgCl to RHE and corrected for ohmic losses. The iR‐corrected potential was calculated as:

(2) $$E_{R\mathrm{HE}}=E_{Ag/Ag\mathrm{Cl}\;}+0.197+0.059\mathrm{pH}+0.95\left|i\right|R$$

where *i* is the cell current and *R* is the uncompensated resistance; the factor 0.95 accounts for 95% iR compensation.

To quantify the relative contributions of kinetics and transport in the polarization behavior, the *j‐E* data were fitted using a mixed kinetic–transport model based on a Koutecký–Levich‐type relationship, in which an exponential kinetic term $j_{k}=j_{0,app}\;\exp\left(-k_{app}E\right)$ is combined in series with an apparent limiting current density *j*_*lim*,*app*_:

(3) $$j=\frac{j_{lim,app}}{1+\left(j_{lim,app}/j_{0,app}\right)\exp\left(k_{app}E\right)}\;$$

Here, *j*_0,*app*_ and *k_app_* describe the low‐current (kinetic‐dominant) regime, whereas *j*_*lim*,*app*_ captures saturation behavior associated with transport limitations. *E* denotes the iR‐corrected cathode potential (V vs. RHE) used as the fitting variable. The transition potentials *E*_0.2_, *E*_1/2_, and *E*_0.8_ (defined as the potentials at which *j* reaches 20%, 50%, and 80% of *j*_*lim*,*app*_, respectively) were calculated from the fitted parameters to compare the onset and progression of transport influence across cathodes. The double‐layer capacitance (C_dl_) was determined from cyclic voltammograms recorded at various scan rates within the non‐Faradaic potential region.

### 4.5 Characterization

SEM was performed using an FEI Inspect F50 field‐emission scanning electron microscope. STEM‐EDS was conducted on an FEI Talos F200X. HR‐TEM was performed using an FEI Titan TM 80–300. N_2_ volumetric adsorption analysis was performed using a Micromeritics ASAP 2020 at 77 K. XRD patterns were obtained by Bruker D8 ADVANCE LynxEye. The Cu content of Cu/K6 was quantified by ICP‐MS. Cu/K6 powder (5 mg) was digested in 6 mL of concentrated HCl in a PTFE‐sealed vessel at 270°C for 2.5 h. After digestion, the solution was diluted with DIW to a final volume of 50 mL. The measured Cu mass was 1.11 mg, corresponding to a Cu content of 22.2 wt.% in Cu/K6. XAS measurements were performed at the 1D and 10D beamlines of the Pohang Accelerator Laboratory (PAL, Korea). Both ex situ and operando experiments were carried out using a flow‐cell configuration adapted for XAS acquisition. A Si (111) channel‐cut monochromator was used to scan the incident energy. Cu K‐edge spectra were collected primarily in fluorescence mode, and transmission data were acquired in parallel when feasible. The energy scale was calibrated using a Cu foil reference, which was repeatedly measured during each beamtime to ensure consistent energy alignment and to correct for potential drift across runs. X‐ray absorption near‐edge structure (XANES) and EXAFS analyses were conducted using the IFEFFIT‐based packages Athena and Artemis. Raw spectra were calibrated, aligned, and normalized following standard procedures, including background subtraction and removal of pre‐edge and post‐edge contributions. EXAFS fitting was carried out in R‐space using theoretical scattering paths generated with FEFF.

### 4.6 Computational Methods

The system of governing equations was solved using the MUMPS direct solver in COMSOL Multiphysics 6.3, with a relative convergence tolerance of 10^−3^. To accurately resolve the sharp concentration gradients, the maximum element size was set to 39.2 nm, which was locally refined down to 0.8 nm in the vicinity of the catalyst layer.

### 4.7 Theory and Model Development

A 2D continuum model was developed for the gas flow channel, gas diffusion layer, catalyst layer, carbon support layer, and electrolyte flow channel of the CO_2_ flow‐cell. The catalyst layer was modeled as pores defined by aggregates of the carbon support, and its properties were determined from experimental results. The full set of governing equations and model parameters is provided in the Supporting Information. The same kinetic parameter set (fit values in Table S5) was used for both the Cu/K6 and Cu+K6 models.

## Author Contributions

**Hyunwook Kim**: conceptualization, investigation, formal analysis, visualization, writing – original draft. **Eunsol Kang**: methodology, investigation, formal analysis, writing – original draft. **Subin Park**: investigation, formal analysis, data curation. **Hyeonggeon Lee**: software, data curation. **Jun Woo Park**: software, data curation. **Dongjin Kim**: investigation, data curation. **Kezia Langie**: investigation, data curation. **Younghyun Chae**: investigation, data curation. **Nara Lee**: investigation, data curation. **Dong‐Joon Lee**: formal analysis, data curation. **Ki Bong Lee**: formal analysis, data curation. **Ung Lee**: methodology, supervision, funding acquisition, writing – review & editing. **Da Hye Won**: conceptualization, supervision, project administration, funding acquisition, writing – review & editing.

## Conflicts of Interest

The authors declare no conflicts of interest.

## Supporting information




**Supporting File**: advs78727‐sup‐0001‐SuppMat.pdf.

## Data Availability

The data that support the findings of this study are available from the corresponding author upon reasonable request.
