# Supplementary material for: Reframing Porous Carbon as a Transport Framework for High‐Rate CO2 Electroreduction
Source: Adv Sci (Weinh). 2026 Nov 10:e78727. Online ahead of print. doi: 10.1002/advs.78727 (PMC13752248; doi:10.1002/advs.78727)
Supplement: Supplementary file 1 [file ADVS-9999-e78727-s001.pdf]

Supporting Information

Reframing Porous Carbon as a Transport Framework for High-Rate CO_2 Electroreduction

Hyunwook Kim[a,b†], Eunsol Kang[a,c,†], Subin Park[a,c], Hyeonggeon Lee[a,c], Jun Woo Park[a], Dongjin Kim[a], Kezia Langie[a,d], Younghyun Chae[a], Nara Lee[a,c], Dong-Joon Lee[c], Ki Bong Lee[c], Ung Lee[e,*], and Da Hye Won[a,f,*]

[a]Clean Energy Research Center, Korea Institute of Science and Technology, Seoul 02792, Republic of Korea

[b]Department of Chemical Engineering, Queen's University, Kingston K7L 3N6, ON, Canada

[c]Department of Chemical and Biological Engineering, Korea University, Seoul 02841, Republic of Korea

[d]Department of Chemistry, Kookmin University, Seoul 02707, Republic of Korea

[e]Department of Chemical and Biomolecular Engineering, Sogang University, Seoul 04107, Republic of Korea

[f]Division of Energy and Environmental Technology, KIST School, University of Science and Technology (UST), Seoul 02792, Republic of Korea

[†]These authors contributed equally as first authors.

*Corresponding authors

E-mail: ulee@sogang.ac.kr (U. Lee), dahye0803@kist.re.kr (D.H. Won)

Governing equations and model description

The current density (i_s) and potential (ϕ_s) profiles in the solid fraction of the catalyst layer were determined using Ohm's law,

$$i_s = -\nabla \cdot (\sigma_{eff}\phi_s) \tag{1}$$

The effective conductivity (σ_{eff}) is estimated based on the nominal conductivity of solid copper σ_s using the Bruggeman relation,

$$\sigma_{eff} = \epsilon_s^{1.5}\sigma_s \tag{2}$$

The active specific surface area (a_v), representing the active surface available for the electrochemical reaction, was derived from the solid volume fraction ($1-\epsilon_{Cu}$) and the mean particle radius (r_{Cu}). The equation incorporates a geometric shape factor of 3, resulting from the surface-to-volume ratio of spherical particles ($\frac{4\pi r^2}{(4/3)\pi r^3} = \frac{3}{r}$).

$$a_v = \frac{3(1-\epsilon_{Cu})}{r_{Cu}} \tag{3}$$

The gas and liquid pressure in the porous media (gas diffusion layer, GDL; catalyst layer, CL; and carbon support layer, CSL) are calculated via mass conservation

$$\nabla \cdot \rho_p u_p = Q_p \tag{4}$$

and Darcy's law,

$$u_p = -\frac{\psi_{m,p}^{eff}}{\mu_p}\nabla p_p \tag{5}$$

u_p represents the mass-averaged velocity field of phase p, $\psi_{m,p}^{eff}$ is the effective permeability of phase p in medium m, ρ_p and μ_p are the fluid density and viscosity respectively, Q is the mass source term.

In the gas phase, CO_2, H_2O, H_2, CO, CH_4, and C_2H_4 are present. The mole fractions are calculated using mole balance,

$$\nabla \cdot N_i = R_{B,i} + R_{CT,i} + R_{PT,i} \tag{6}$$

here, $R_{B,i}, R_{CT,i}$ and $R_{PT,i}$ denote the molar source terms of species i due to bulk homogeneous reactions, charge transfer reactions, and phase-transfer reactions, respectively. The molar flux(N_i) is derived from the mass flux,

$$N_i = \frac{1}{M_i}\left(-\rho_G D_i^{eff}\nabla\omega_i - \rho_G D_i^{eff}\omega_i\frac{\nabla M_A}{M_A} + \rho_i u_G\right. \tag{7}$$

Where ρ_G is the density of gaseous mixture, ω_i is the mass fraction, M_i, D_i^{eff} is the molecular weight and effective diffusivity of species i respectively. In addition, the sum of mole fractions,

$$\Sigma_i x_i = 1 \tag{8}$$

where the mole fraction $x_i = \frac{\omega_i M_A}{M_i}$.

In the ionomer phase, charged species OH^-, HCO_3^-, CO_3^{2-}, H^+, K^+, and neutral species H_2O, CO_2(aq) exist in the CL, CSL and electrolyte flow channel (EFC). Due to their low solubility, gaseous species H_2, CO, and C_2H_4 are neglected. The molar flux of these species is described by the Nernst-Planck equation for dilute-solutions,

$$N_j = -D_j^{eff} \nabla c_j - \frac{z_j F}{RT} D_f^{eff} c_j \nabla \phi_L \tag{9}$$

where D_j^{eff}, z_j, c_j are the effective diffusion coefficient, charge, and concentration of species j, respectively, and F is Faraday's constant (96485 C/mol). To solve the electrolyte ionic potential, electroneutrality is needed.

Bulk reactions are obtained from suitable rate expressions. The volumetric molar source term, $R_{B,j}$, represents the contributions of homogeneous reactions taking place within the ionomer and membrane domains, encompassing the following.

$$CO_2(aq) + H_2O \xleftrightarrow{k_1, k_{-1}} H^+ + HCO_3^- \tag{10}$$

$$HCO_3^- \xleftrightarrow{k_2, k_{-2}} H^+ + CO_3^{2-} \tag{11}$$

$$CO_2(aq) + OH^- \xleftrightarrow{k_3, k_{-3}} HCO_3^- \tag{12}$$

$$HCO_3^- + OH^- \xleftrightarrow{k_4, k_{-4}} H_2O + CO_3^{2-} \tag{13}$$

$$H_2O \xleftrightarrow{k_w, k_{-w}} H^+ + OH^- \tag{14}$$

$$HCOOH \xleftrightarrow{k_5, k_{-5}} H^+ + HCOO^- \tag{15}$$

Here, k_n and k_{-n} represent the forward and reverse rate constants for the homogeneous reaction n, respectively, while K_n denotes the equilibrium constant. Consequently, the volumetric source term $R_{B,j}$ is formulated as follows.

$$R_{B,j} = \sum_n s_{j,n} c^{ref} \left(k_n \prod_{s_{j,n}<0} a_j^{-sj,n} - \frac{k_n}{K_n} \prod_{s_{j,n}>0} a_j^{s_{j,n}} \right) \tag{16}$$

where $s_{j,n}$ corresponds to the stoichiometric coefficient of species j in reaction n, taking a negative value for reactants and a positive value for products.

The electrochemical performance of the system is governed by the kinetics of the cathode reactions occurring at the catalyst-electrolyte interface. The model explicitly incorporates a comprehensive set of nine electrochemical reactions, comprising the reduction of CO_2 into various C_1 and C_{2+} products, as well as the competing hydrogen evolution reaction (HER).

The specific reaction pathways and their corresponding standard electrode potentials ($U^0 vs. SHE$) considered in this study are listed in Equations. (17) – (25).

$$CO_2(aq) + H_2O + 2e^- \longrightarrow CO + 2OH^- \tag{17}$$

$$CO_2 + H_2O + 2e^- \longrightarrow HCOO^- + OH^- \tag{18}$$

$$2H_2O + 2e^- \longrightarrow H_2 + 2OH^-(HER) \tag{19}$$

$$2CO_2 + 8H_2O + 12e^- \longrightarrow C_2H_4 + 12OH^- \tag{20}$$

$$2CO_2 + 9H_2O + 12e^- \longrightarrow C_2H_5OH + 12OH^- \tag{21}$$

$$CO_2 + 6H_2O + 8e^- \longrightarrow CH_4 + 8OH^- \tag{22}$$

$$2CO_2 + 5H_2O + 8e^- \longrightarrow CH_3COO^- + 7OH^- \tag{23}$$

$$2CO_2 + 10H_2O + 14e^- \longrightarrow C_2H_6 + 14OH^- \tag{24}$$

$$3CO_2 + 13H_2O + 18e^- \longrightarrow C_3H_7OH + 18OH^- \tag{25}$$

The source term accounting for charge transfer reactions, $R_{CT,j}$, is exclusively applied within the CL domains and is expressed as a function of the partial current densities corresponding to reactions (17) – (25).

$$R_{CT,j} = -\sum_k \frac{s_{j,k} a_v i_k}{n_k F} \tag{26}$$

Here, n_k represents the number of electrons transferred in reaction k, and a_v is the specific active surface area as defined in Equation (3). The local partial current density, i_k is governed by reaction kinetics described by the Butler-Volmer equation:

$$i_k = i_{o,k} \prod_j \left(\frac{c_j}{c_j^{ref}}\right)^{\gamma_{j,k}} \cdot exp\left(\frac{\alpha_{c,k} F}{RT} \eta_k\right) \tag{27}$$

In this expression, $i_{o.k}$ denotes the exchange current density for reaction k. c_j and c_j^{ref} represent the local surface concentration and the reference concentration of species j, respectively, with $\gamma_{j,k}$ denoting the reaction order. Furthermore, $\alpha_{c,k}$ is the cathodic transfer coefficient. The kinetic driving force is quantified by the overpotential, η_k, which is defined as the deviation of the local electric potential difference from the thermodynamic equilibrium potential, adjusted for local pH variations:

$$\eta_k = (\phi_s - \phi_L) - \left(U_k^o - \frac{2.303RT}{F} pH\right) \tag{28}$$

where ϕ_s and ϕ_L denote the electric potentials of the solid conducting phase and the liquid electrolyte, respectively, and U_k^o is the standard equilibrium, potential for reaction k.

Subsequently, the term $R_{PT,j,p}$ characterizes the mass transfer rate of species such as CO_2 across the interfaces (gas | ionomer, liquid | ionomer, and gas | liquid) towards phase p.

$$R_{PT,j,I} = a_s k_{MT,j}\left(c_j^{eq} - c_j\right) \tag{29}$$

where $k_{MT,j}$ denotes the mass transfer coefficient, and c_j^{eq} is the equilibrium concentration of species j relative to its concentration outside the ionomer.

To accurately capture the gas transport phenomena within the porous domains, the diffusion coefficient for each gaseous species i was calculated by considering two distinct mechanisms: bulk molecular diffusion and Knudsen diffusion.

First, the Stefan-Maxwell diffusivity (D_i^{SM}) was employed to account for multi-component molecular interactions in the gas mixture. This is defined using the mass-averaged formulation:

$$D_i^{SM} = \frac{1-\omega_i}{\sum_{q\neq i}\frac{y_q}{D_{iq}}} \tag{30}$$

where ω_i represents the mass fraction of species i, y_q is the mole fraction of species q, and D_{iq} denotes the binary diffusion coefficient derived using Fuller et al., which explicitly accounts for the molecular properties and operating conditions:

$$D_{iq} = \frac{10^{-3} T[K]^{1.75}\left(M_i[gmol^{-1}]^{-1} + M_q[gmol^{-1}]^{-1}\right)^{0.5}}{p[atm]\left(\nu_{p,i}^{0.33} + \nu_{p,q}^{0.33}\right)^2} \tag{31}$$

In this equation, M denotes the molar mass, p is the pressure in atm, and ν_p represents the atomic diffusion volume of each species.

Additionally, to address transport limitations arising from the confinement of molecules within narrow pores, the Knudsen diffusivity (D_i^K) was introduced. This term describes the collisions between gas molecules and the pore walls and is calculated as:

$$D_i^K = \frac{2r_{pore,m}}{3}\sqrt{\frac{8RT}{\pi M_i}} \tag{32}$$

here, $r_{pore,m}$ is the average pore radius of the medium m.

Finally, the effective diffusion coefficient (D_i) combines these two mechanisms in parallel, assuming that both resistance paths act simultaneously. It is determined by the harmonic mean of the Stefan-Maxwell and Knudsen diffusivities:

$$D_i = \left(\frac{1}{D_i^{SM}} + \frac{1}{D_i^K}\right)^{-1} \tag{33}$$

To determine the water activity within the catalyst layer, the mole fraction of water(x_w) was explicitly calculated from the water content (λ). The conversion is governed by the following relationships:

$$x_w = \frac{\lambda}{\lambda + 1} \tag{34}$$

where λ represents the number of water molecules per fixed charge group in the ionomer. The relationship between water activity and water content was adopted from literature data for the Fumasep FAA-3 membrane.

Supplementary Note

Cu K-edge X-ray absorption near-edge structure (XANES) was used to evaluate the Cu oxidation states of Cu/C samples (Cu/VC, Cu/K3, and Cu/K6) and the physically mixed Cu+K6 benchmark using Cu foil, Cu2O, and CuO as references (Figures S7 and S8). Cu+K6 exhibited a spectrum close to Cu foil, whereas linear combination fitting of Cu/VC, Cu/K3, and Cu/K6 revealed a progressively more reduced average Cu state from Cu/VC to Cu/K6. The Fourier-transformed extended X-ray absorption fine structure (EXAFS) and Cu L-edge spectra showed consistent trends (Figures S9 and S10). Together with the high-resolution transmission electron microscopy (HR-TEM) and X-ray diffraction (XRD) results, these observations indicate mixed Cu^0/Cu^+ nanodomains in the Cu/C catalysts, with minor Cu(II)-like contributions detectable by XAS.

Operando Cu K-edge EXAFS further showed that the Cu/C catalysts evolved toward predominantly metallic-like coordination under cathodic CO_2RR conditions (Figure S11). A clear Cu–Cu contribution emerged upon cathodic polarization and remained dominant with increasing current density. Cu+K6 similarly retained predominantly metallic-like coordination during operation, as shown in Figure 3e of the main text. Thus, differences in the initial Cu oxidation state become less pronounced under CO_2RR conditions.

Table S1. Textural properties of the carbon supports derived from N_2 adsorption–desorption at 77 K.

Porous carbon	BET surface area (S_{BET}) ($m^2\ g^{-1}$)	Total pore volume (V_T) [1] ($cm^3\ g^{-1}$)	Micropore volume (V_m) [2] ($cm^3\ g^{-1}$)	Mesopore volume ($V_M=V_T-V_m$) ($cm^3\ g^{-1}$)	V_M/V_m	Pore size [3] (nm)
VC	280	0.35	0.11	0.24	2.2	9.4
K3	705	0.84	0.30	0.54	1.8	5.8
K6	1245	2.38	0.53	1.85	3.5	8.0

[1] V_T measured at $p/p_0 \approx 0.99$.

[2] V_m determined by the HK method.

[3] Average pore size estimated from the BJH desorption method.

Table S2. Textural properties of the Cu/C catalysts derived from N_2 adsorption–desorption at 77 K.

Cu/C catalysts	BET surface area (S_{BET}) (m^2 g^{-1})	Total pore volume (V_T) [1] (cm^3 g^{-1})	Micropore volume (V_m) [2] (cm^3 g^{-1})	Mesopore volume (V_M=V_T−V_m) (cm^3 g^{-1})	V_M/V_m	Pore size [3] (nm)
Cu/VC	121	0.26	0.05	0.21	4.2	16.8
Cu/K3	403	0.59	0.17	0.42	2.5	7.9
Cu/K6	641	1.25	0.27	0.98	3.6	7.0

[1] V_T measured at $p/p_0 \approx 0.99$.

[2] V_m determined by the HK method.

[3] Average pore size estimated from the BJH desorption method.

Table S3. Mixed-control fitting parameters for Cu/K6 and Cu+K6.

Catalysts	$j_{0,app}$ (mA cm^{-2})	k_{app} (V^{-1})	$j_{lim,app}$ (mA cm^{-2})	$E_{0.2 j_{lim}}$ (V)	$E_{1/2}$ (V)	$E_{0.8 j_{lim}}$ (V)	$\Delta E_{0.8-0.2}$ (V)
Cu/K6	0.888	6.29	398	−0.751	−0.971	−1.192	0.441
Cu+K6	0.817	5.93	316	−0.770	−1.004	−1.237	0.467

Table S4. Summary of boundary conditions

Parameter	Cathode GDL | CL	Cathode CL | EFC
ϕ_s	V_{cell}	0 V
p	1 atm	
ω_i	$\omega_{CO_2} = 100$	$\omega_{KOH} = 5.4$
ϕ_L	$\nabla\phi_L = 0$	$\nabla\phi_L = 0$
c_j	$\nabla \cdot N_j = 0$	$\nabla \cdot N_j = 0$
μ_0	$\nabla \cdot N_w = 0$	$\nabla \cdot N_w = 0$

Table S5. 2D continuum model parameters

Parameter	Value	Unit	Reference
CL Properties			
L_{CL}	200	nm	Experiment
H_{CL}	50	nm	Experiment
ϵ_{Cu}	0.2227	1	Experiment
r_{Cu}	20	nm	Experiment
σ_s	$5.8e^{7}$	S/m	Ref. S1
CSL Properties			
L_{CSL}	200	nm	Experiment
H_{CSL}	200	nm	Experiment
ϵ_{CSL}	0.36	1	Experiment
GDL Properties			
L_{GDL}	325	μm	Experiment
ϵ_{DM}	0.526	1	Experiment
Homogeneous Reactions			
k_1	$3.71e^{-2}$	$1/s$	Ref. S2
k_{-1}	$2.67e^{4}$	$L/mol/s$	Ref. S2
k_2	59.44	$1/s$	Ref. S2
k_{-2}	$5.0e^{10}$	$L/mol/s$	Ref. S2
k_3	$2.23e^{3}$	$L/mol/s$	Ref. S2
k_{-3}	$9.71e^{-5}$	$1/s$	Ref. S2
k_4	$6.0e^{9}$	$L/mol/s$	Ref. S2
k_{-4}	$3.06e^{5}$	$1/s$	Ref. S2
k_w	$1.4e^{-3}$	$mol/L/s$	Ref. S2
k_{-w}	$2.31e^{-10}$	$L/mol/s$	Ref. S2
Electrochemical Reaction			
U_{CO}^{0}	-0.11	V	
$i_{0,CO}$	3.8	mA/cm^2	Fit value
$\alpha_{c,CO}$	0.292	1	Fit value

γ_{CO}	1.665	1	Fit value
U^0_{HER}	0	V	Ref. S3
$i_{0,HER}$	52	mA/cm^2	Fit value
$\alpha_{c,HER}$	0.062	1	Fit value
$U^0_{C_2H_4}$	0.07	V	Ref. S3
i_{0,C_2H_4}	3.9	mA/cm^2	Fit value
α_{c,C_2H_4}	0.268	1	Fit value
$\gamma_{C_2H_4}$	4.7	1	Fit value
Liquid phase transport			
$D_{H^+,w}$	$9.3e^{-9}$	m^2/s	Ref. S3
$D_{CO_{2,w}}$	$2.1e^{-9}$	m^2/s	Ref. S3
$D_{OH^-,w}$	$5.3e^{-9}$	m^2/s	Ref. S3
$D_{HCO_3^-}$	$1.18e^{-9}$	m^2/s	Ref. S3
D_{K^+}	$1.96e^{-9}$	m^2/s	Ref. S3

Fit value: electrochemical kinetic parameters obtained by fitting the simulated partial current densities to the flow-cell data. The same values were used for the Cu/K6 and Cu+K6 models, including the MEA-relevant calculations (Figures S31 and S32).

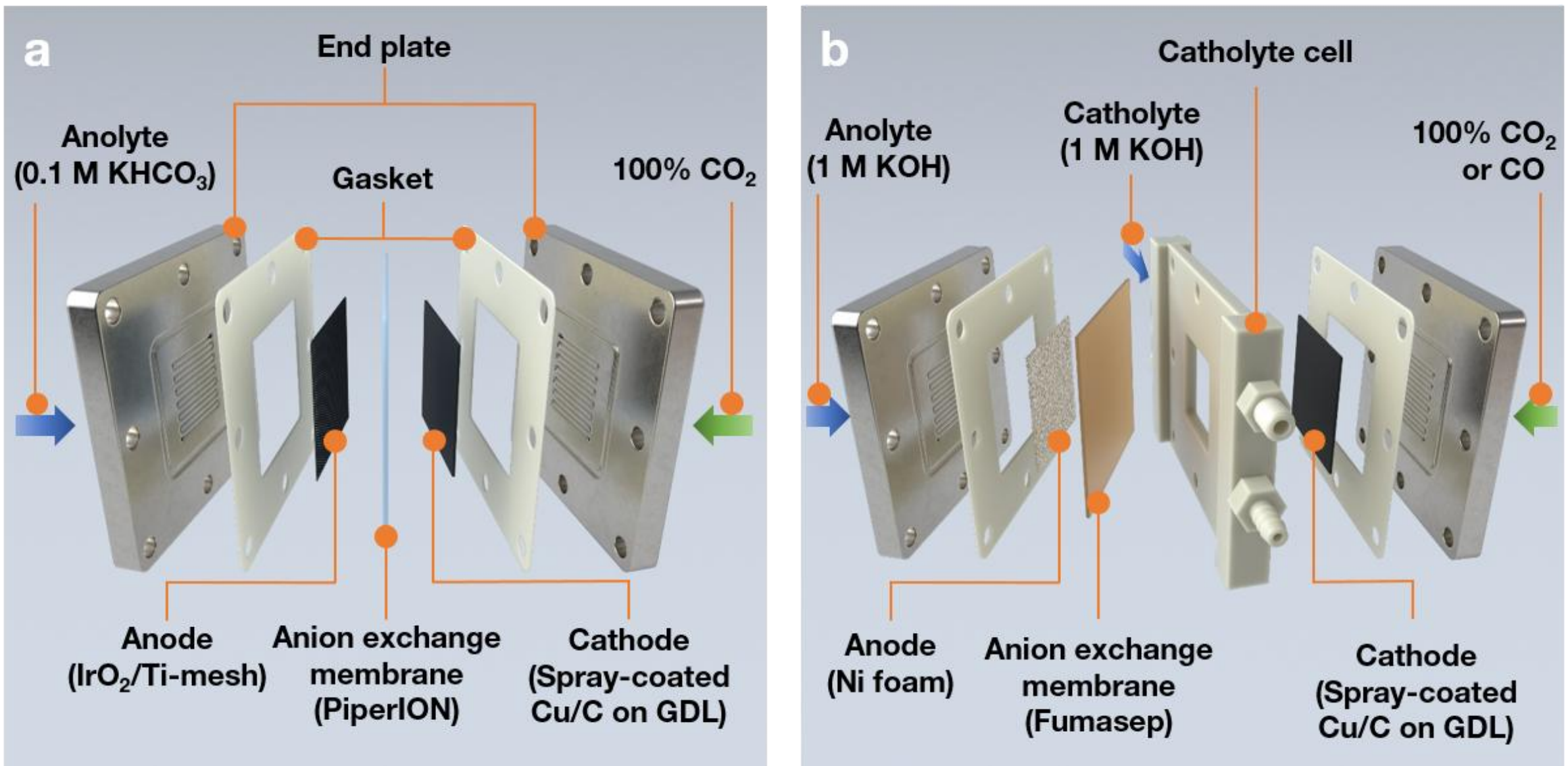

Figure S1. Schematic illustrations of (a) MEA and (b) flow-cell configurations.

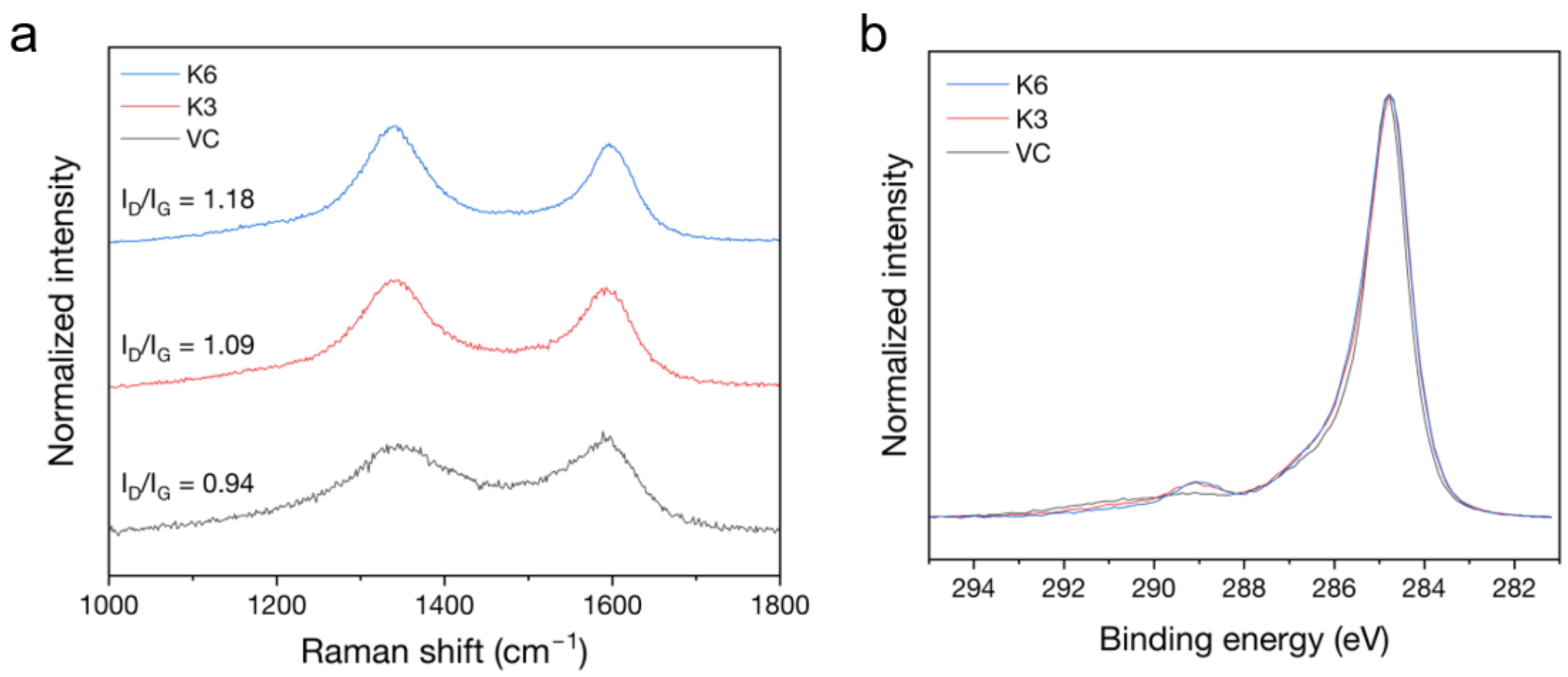

Figure S2. (a) Raman spectra with the corresponding I_D/I_G ratios and (b) C 1s XPS spectra of VC, K3, and K6.

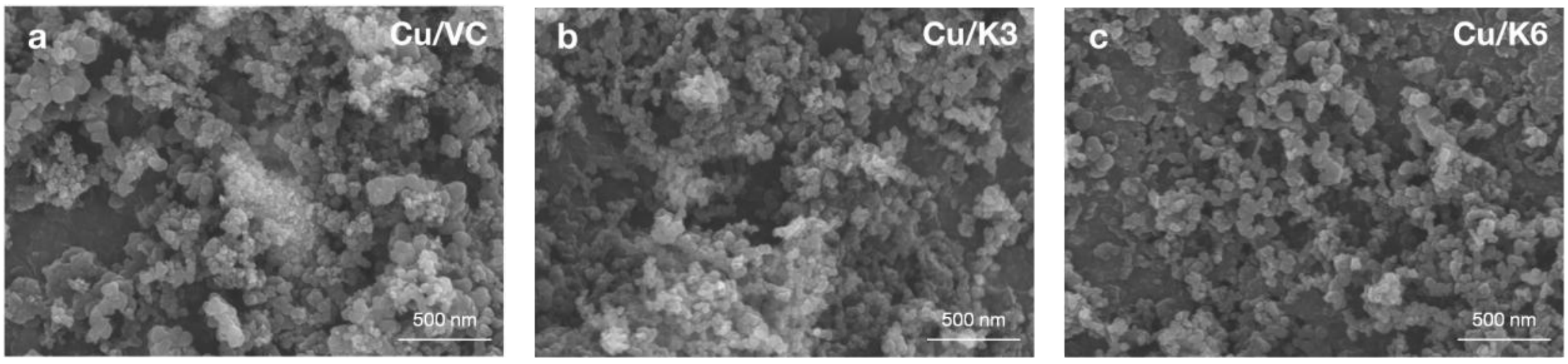

Figure S3. SEM images of Cu/C catalysts prepared using porous carbon as support: (a) Vulcan (VC; Cu/VC), (b) Ketjen Black EC–300J (K3; Cu/K3), and (c) Ketjen Black EC–600JD (K6; Cu/K6).

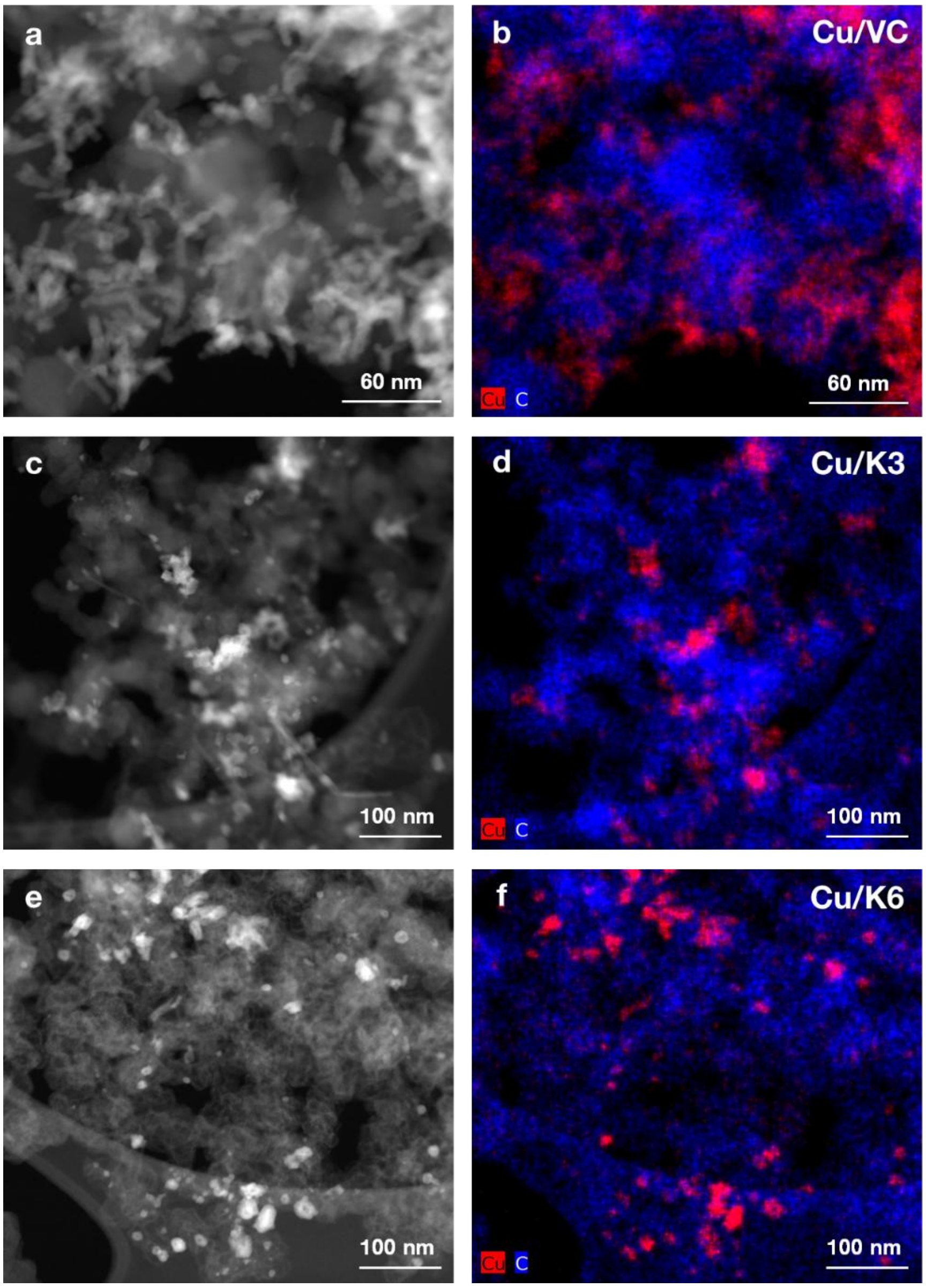

Figure S4. STEM and corresponding EDS elemental maps of (a,b) Cu/VC, (c,d) Cu/K3, (e,f) Cu/K6, showing the spatial distribution of Cu (red) and C (blue).

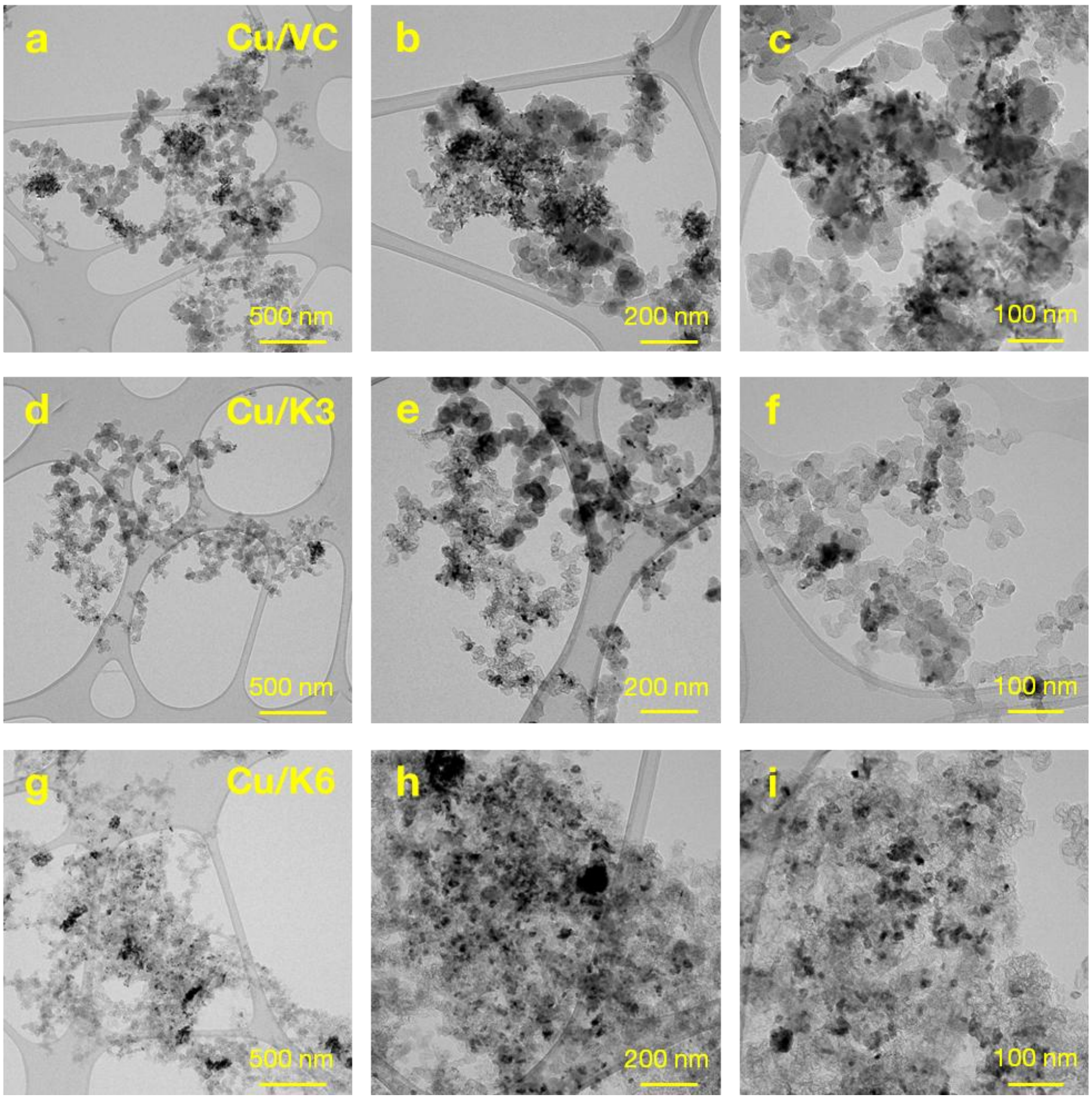

Figure S5. TEM images of Cu/C catalysts at increasing magnification: (a–c) Cu/VC, (d–f) Cu/K3, and (g–i) Cu/K6. Higher-contrast regions indicate Cu-rich domains on the carbon supports.

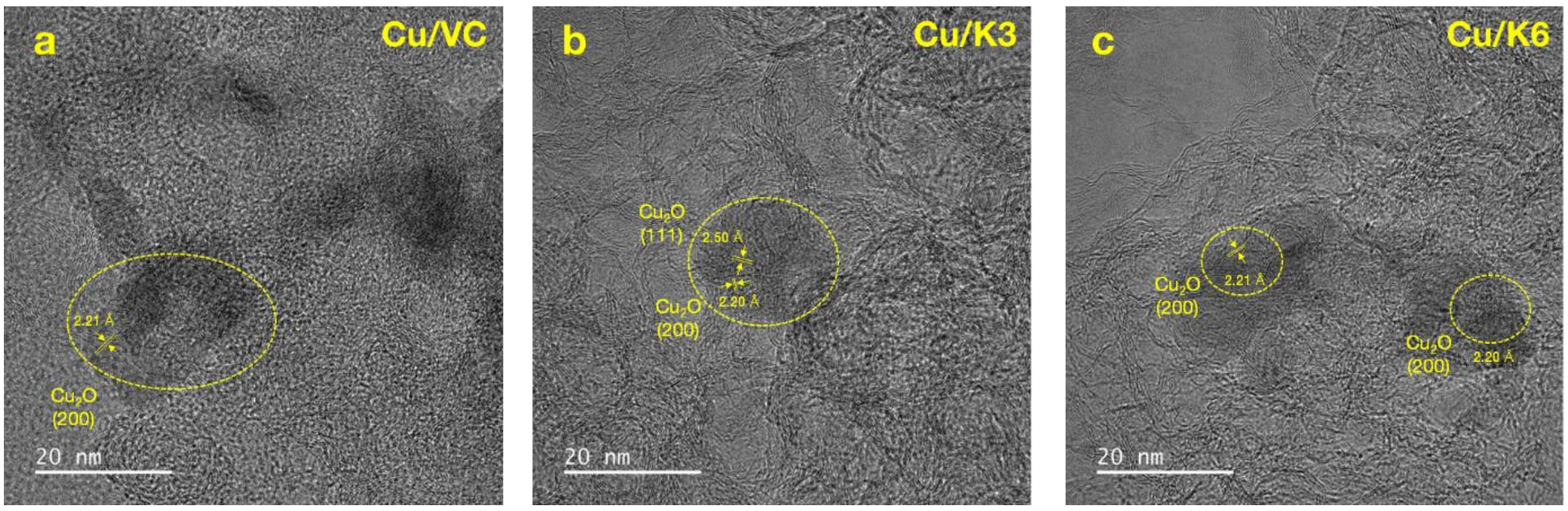

Figure S6. HR-TEM images of (a) Cu/VC, (b) Cu/K3, and (c) Cu/K6, highlighting crystalline Cu-containing domains. Measured lattice fringes are consistent with Cu_2O, including d ≈ 2.20–2.21 Å assignable to Cu_2O (200) and d ≈ 2.50 Å assignable to Cu_2O (111).

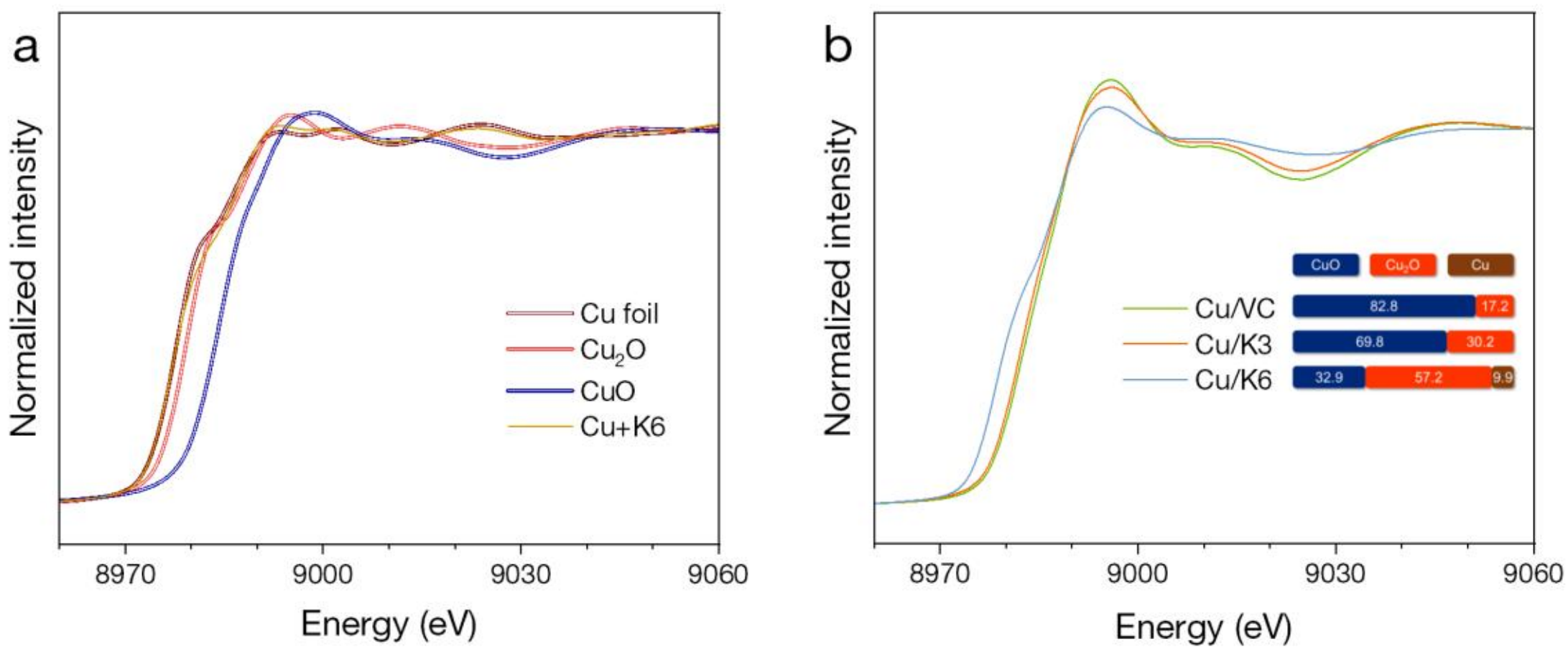

Figure S7. Cu K-edge XANES spectra. (a) Reference spectra of Cu foil, Cu_2O, and CuO compared with the Cu+K6 sample. (b) Normalized Cu K-edge XANES spectra of Cu/VC, Cu/K3, and Cu/K6 with linear combination fitting results using CuO, Cu_2O, and Cu references (inset, %).

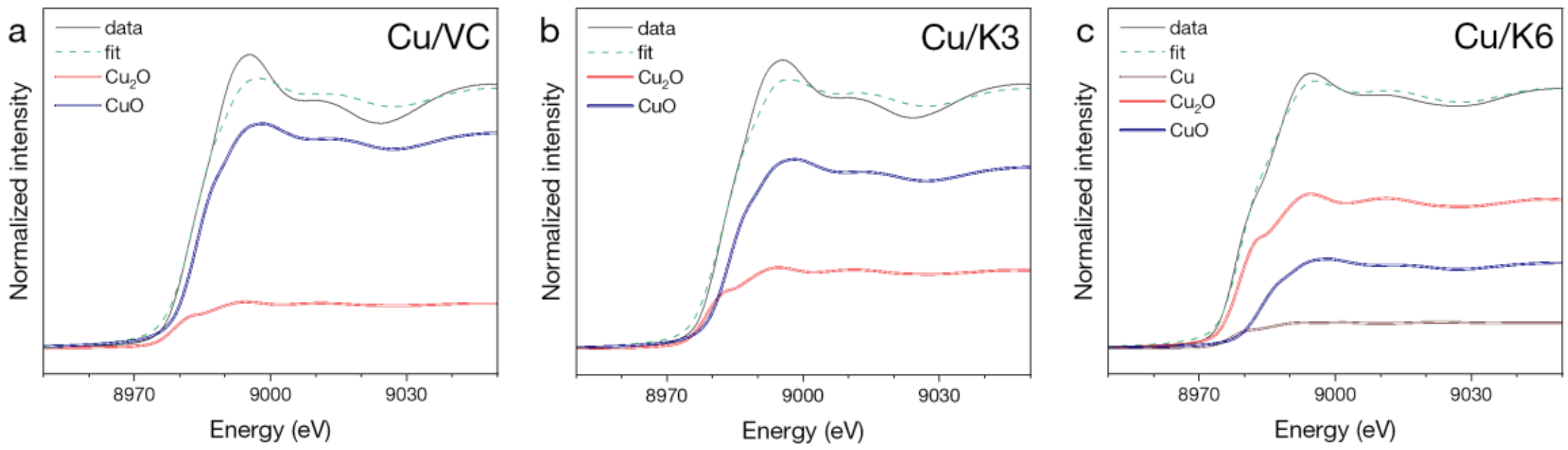

Figure S8. Linear combination fitting of normalized Cu K-edge XANES spectra. Experimental data (black) and best-fit curves (green dashed) obtained using CuO and Cu_2O reference spectra for (a) Cu/VC and (b) Cu/K3, and using Cu, Cu_2O, and CuO references for (c) Cu/K6.

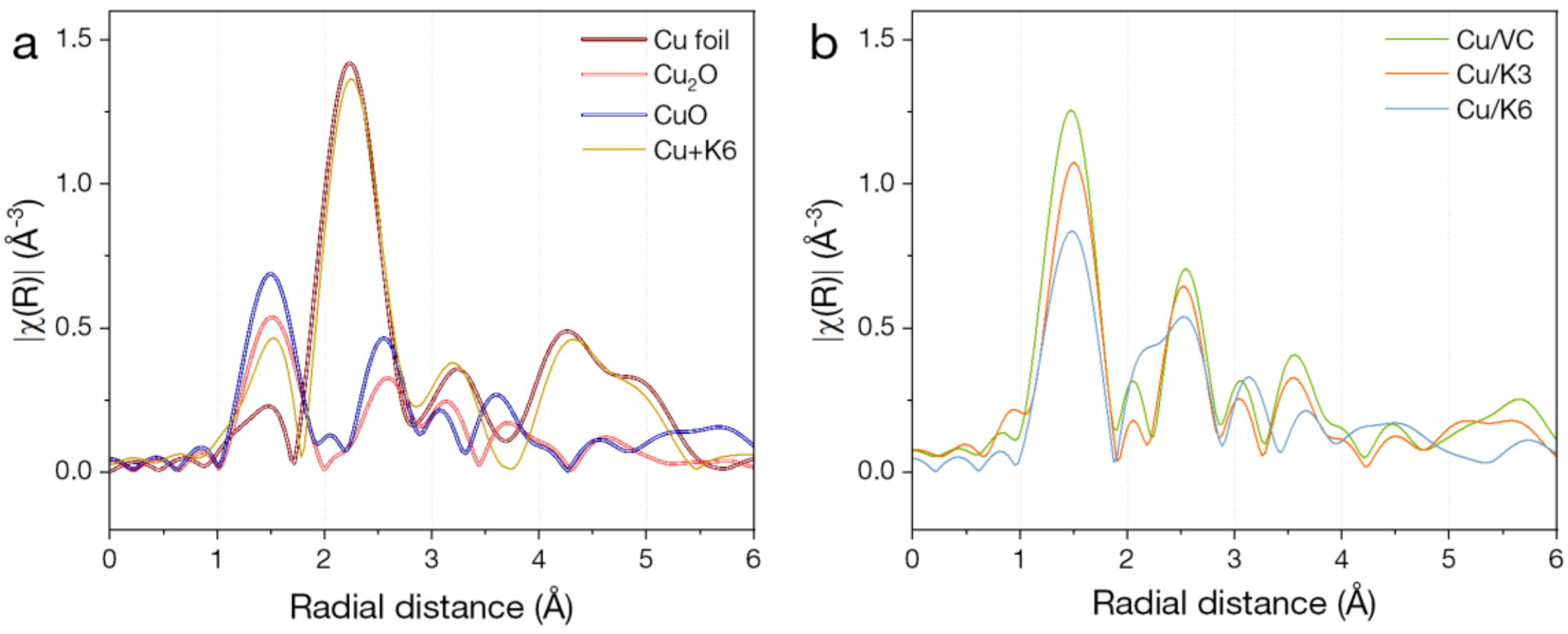

Figure S9. Fourier-transformed Cu K-edge EXAFS spectra shown as |χ(R)|. (a) Reference Cu foil, Cu_2O, and CuO spectra compared with the Cu+K6 sample. (b) |χ(R)| spectra of Cu/VC, Cu/K3, and Cu/K6.

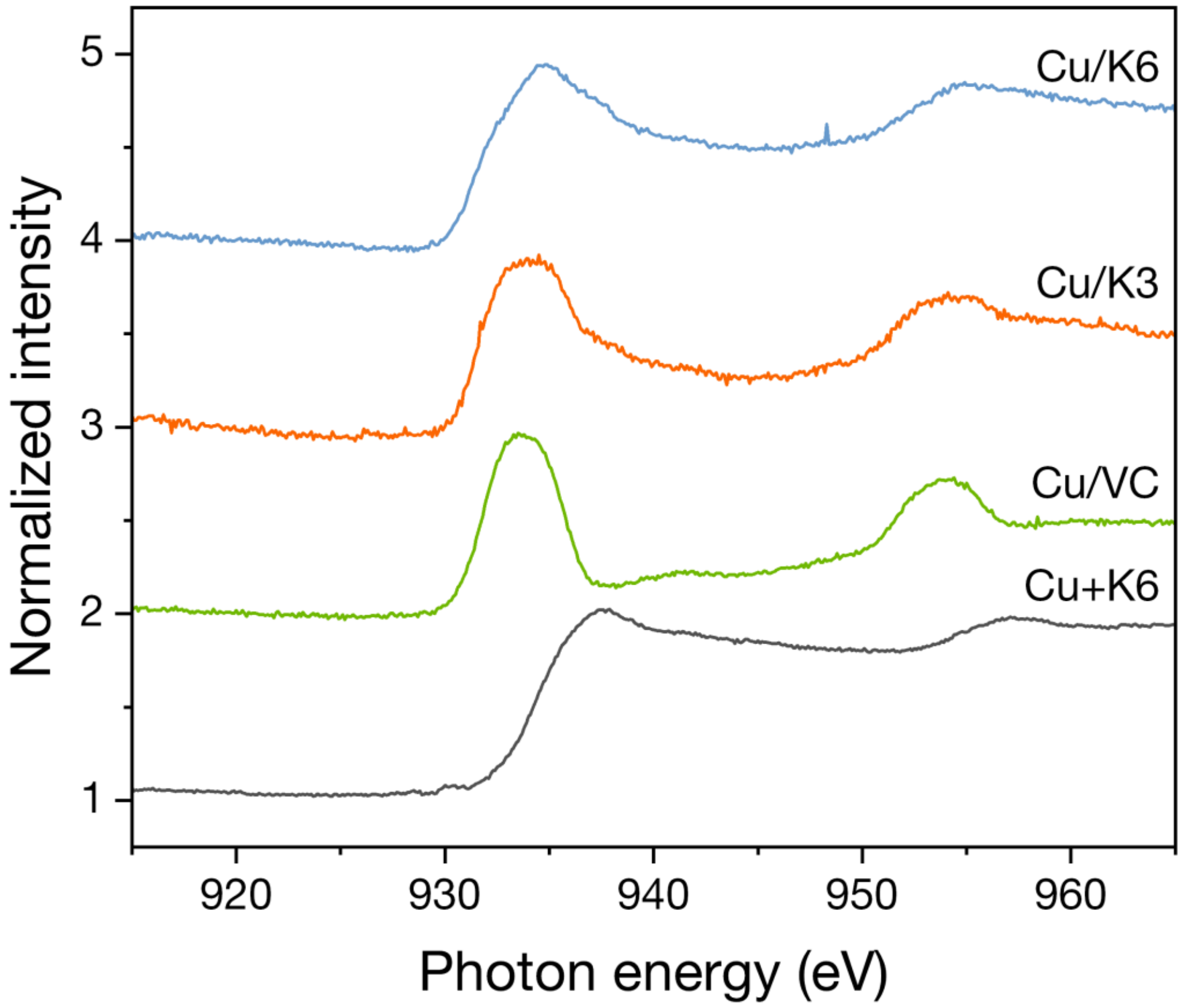

Figure S10. Cu L-edge soft X-ray absorption spectra of Cu/C catalysts (Cu/VC, Cu/K3, and Cu/K6) and Cu+K6.

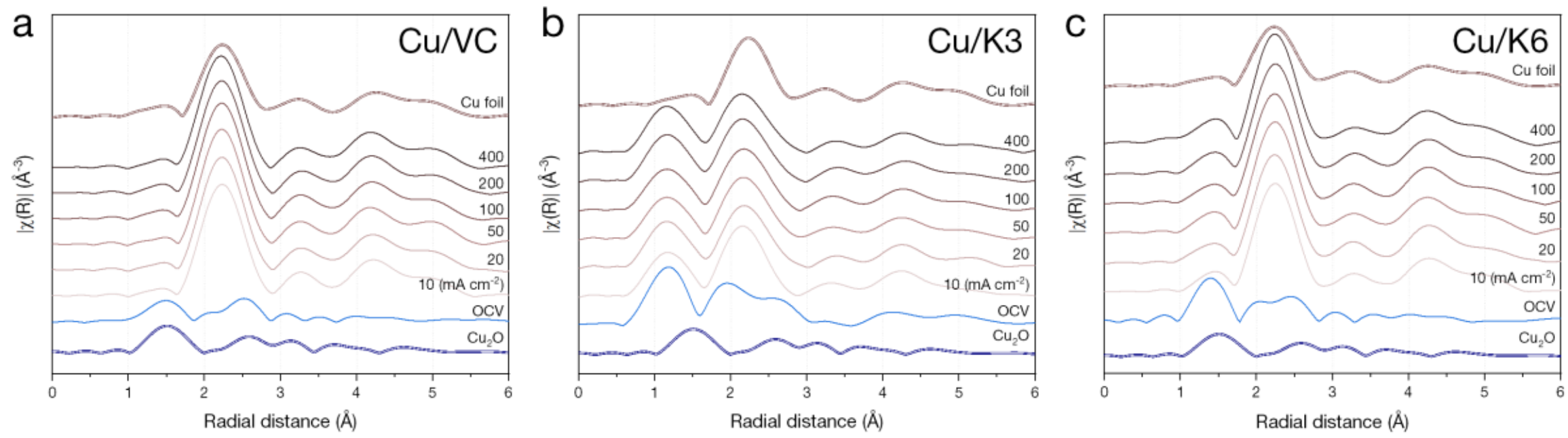

Figure S11. Operando Cu K-edge EXAFS (Fourier-transformed |χ(R)|) of (a) Cu/VC, (b) Cu/K3, and (c) Cu/K6 acquired under CO_2RR, increasing cathodic current density (10–400 mA cm^{-2}). Spectra collected at open-circuit voltage (OCV) are shown in blue; reference spectra of Cu_2O (navy) and Cu foil (top trace) are included for comparison.

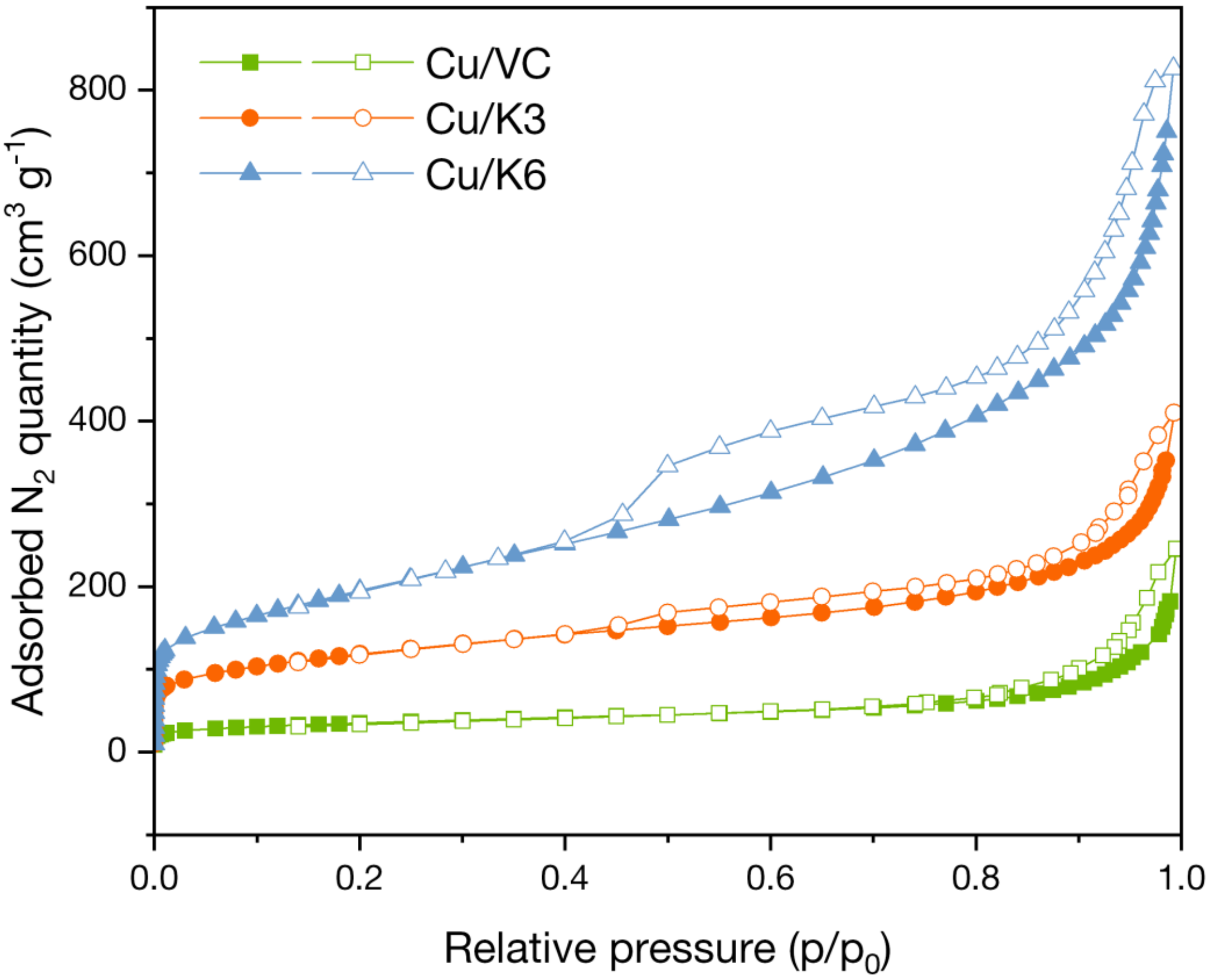

Figure S12. N_2 adsorption–desorption isotherms of Cu/C catalysts (Cu/VC, Cu/K3, and Cu/K6) measured at 77 K. Filled and open symbols denote adsorption and desorption branches, respectively.

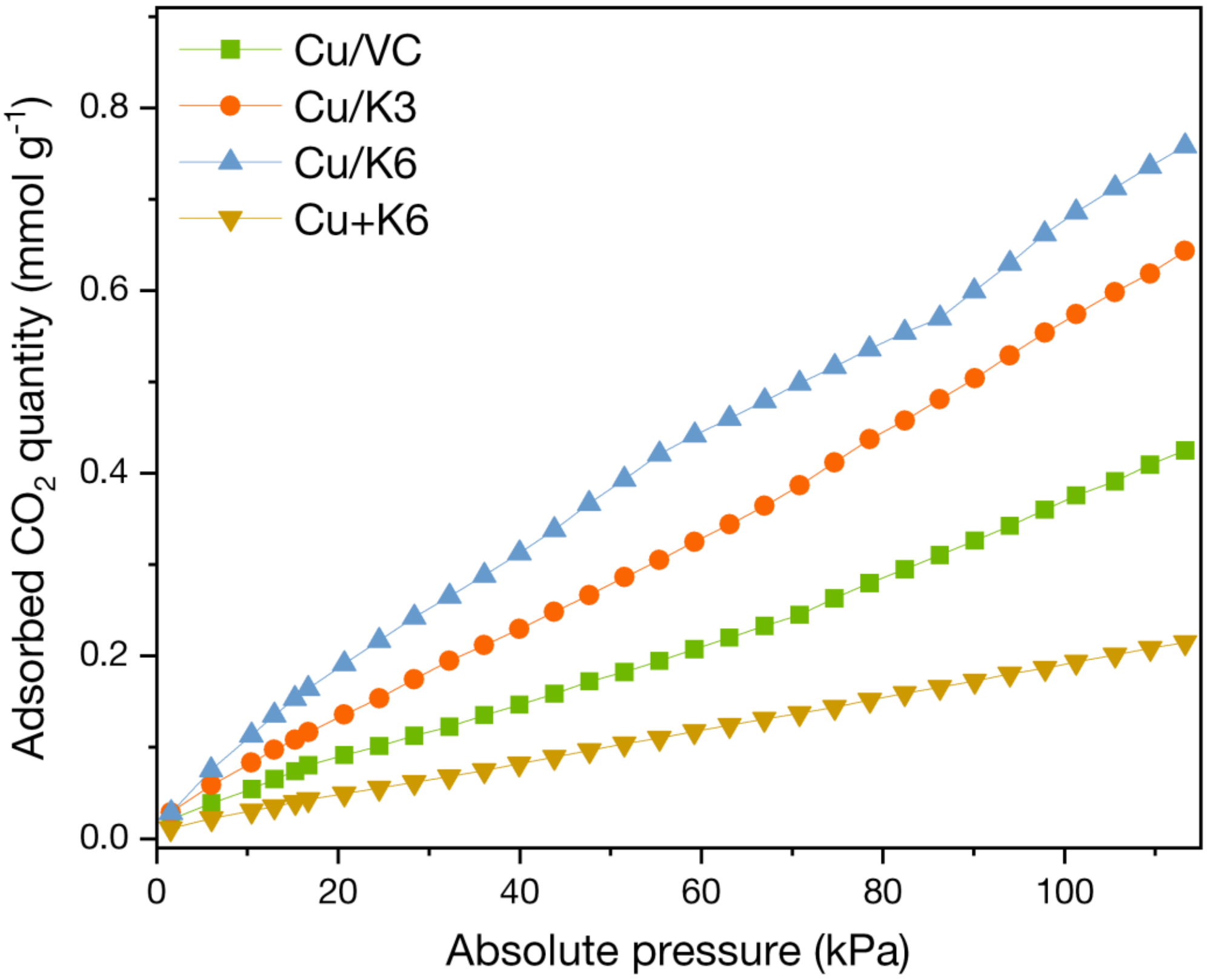

Figure S13. CO_2 adsorption isotherms of Cu/C catalysts (Cu/VC, Cu/K3, and Cu/K6) and Cu+K6 at 25 °C.

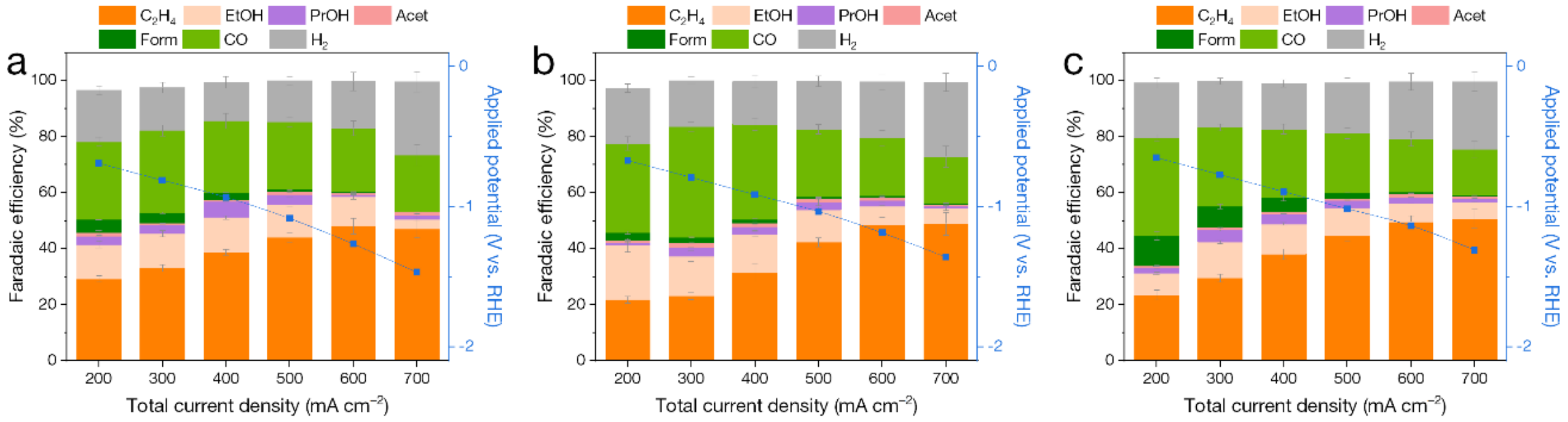

Figure S14. Product distribution during CO_2RR in the flow-cell setup as a function of total current density for (a) Cu/VC, (b) Cu/K3, and (c) Cu/K6. Stacked bars show Faradaic efficiencies, and the blue line (right axis) denotes the corresponding applied potential (V vs. RHE).

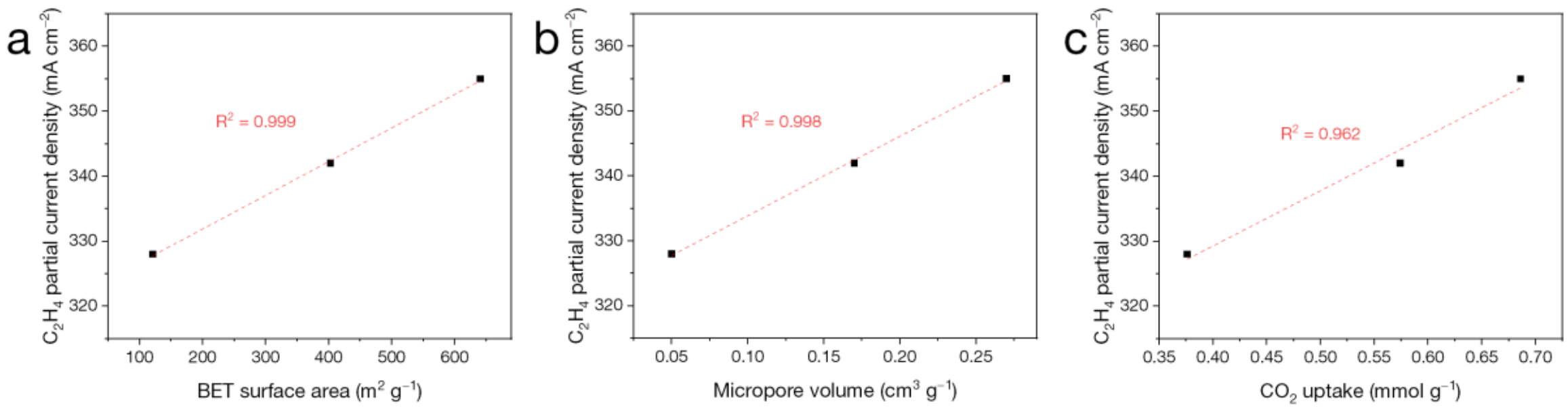

Figure S15. Correlation between C_2H_4 partial current density and support textural/adsorption descriptors. C_2H_4 partial current density plotted as a function of (a) BET surface area, (b) micropore volume, and (c) CO_2 uptake. Dashed red lines indicate linear fits; R^2 values are shown in each panel.

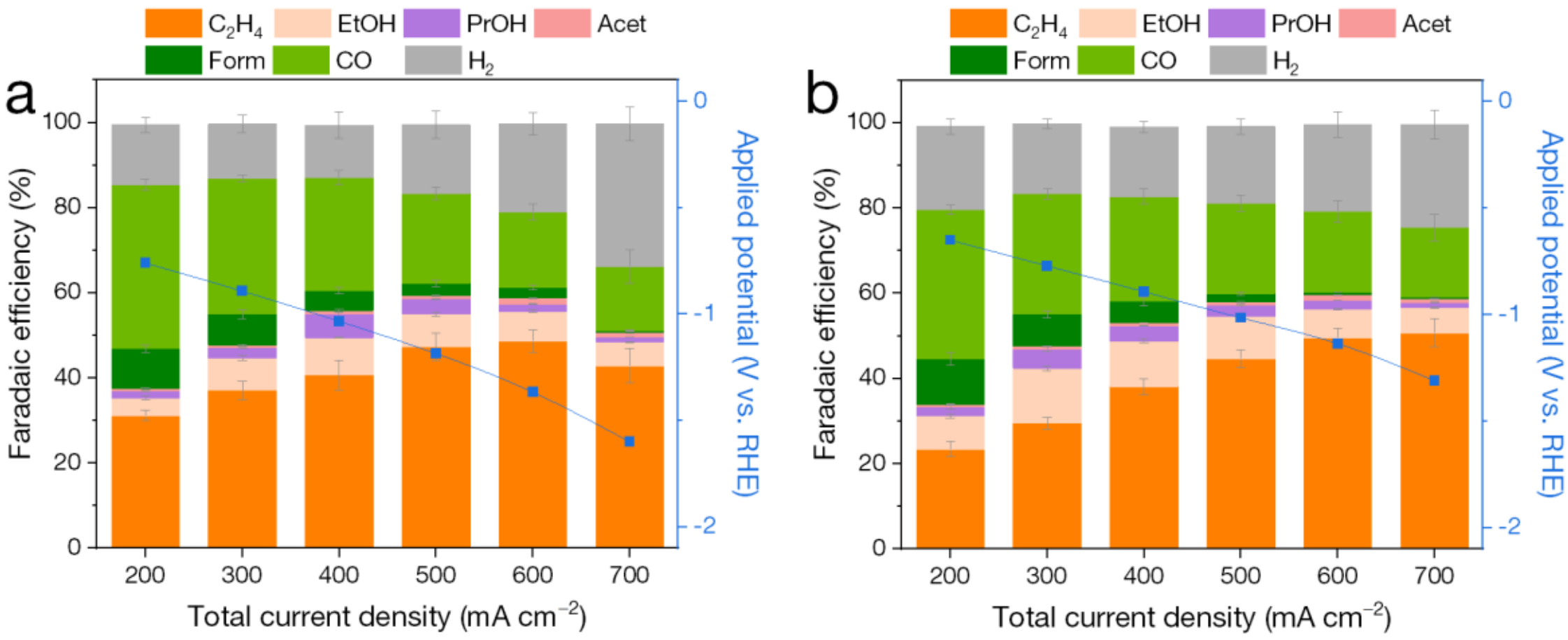

Figure S16. Product distribution during CO₂RR in the flow-cell setup as a function of total current density for (a) Cu+K6 and (b) Cu/K6. Stacked bars show Faradaic efficiencies, and the blue line (right axis) denotes the corresponding applied potential (V vs. RHE).

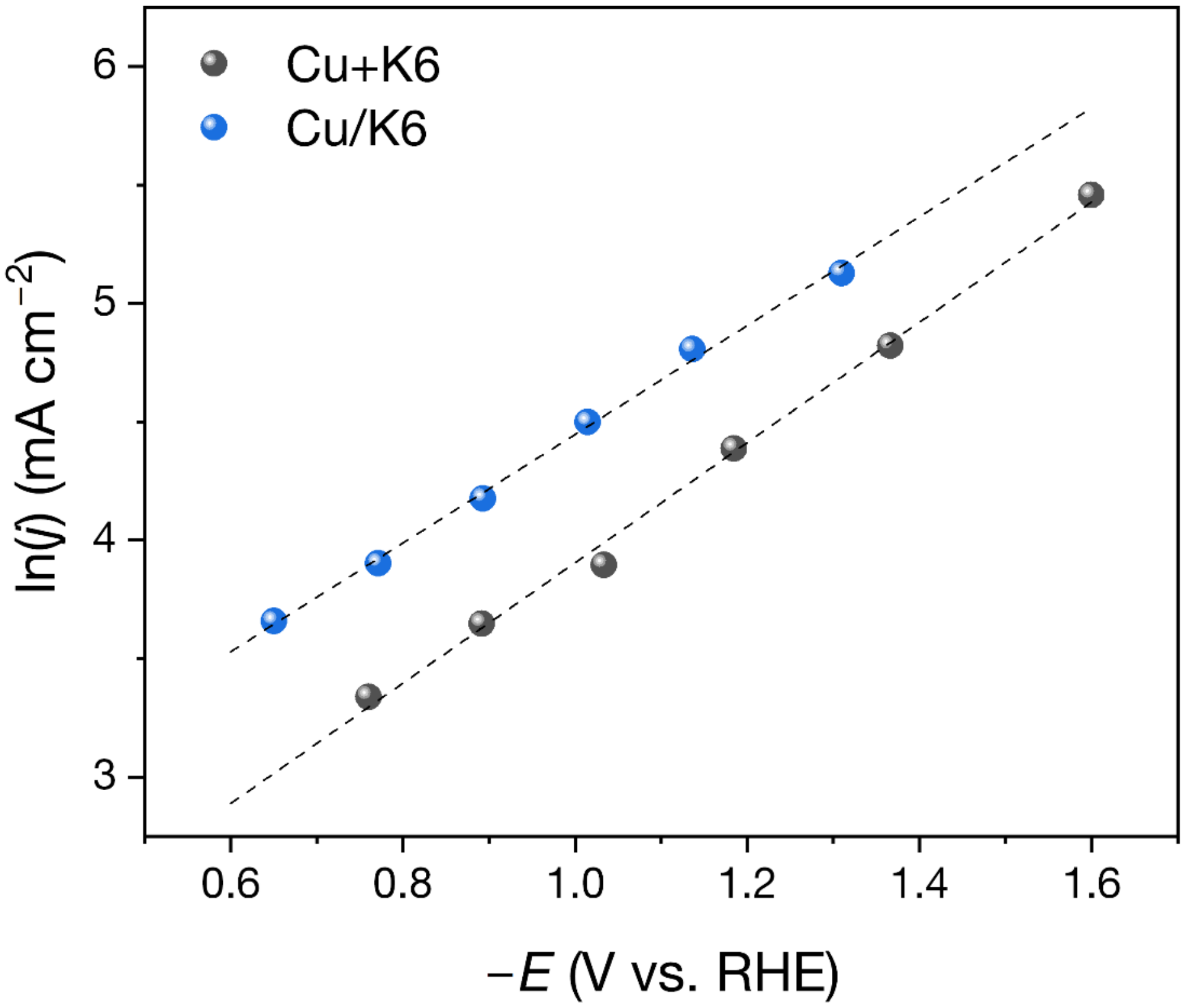

Figure S17. Mixed kinetic−transport analysis of j_{H2} plotted versus −*E* from flow-cell CO_2RR data.

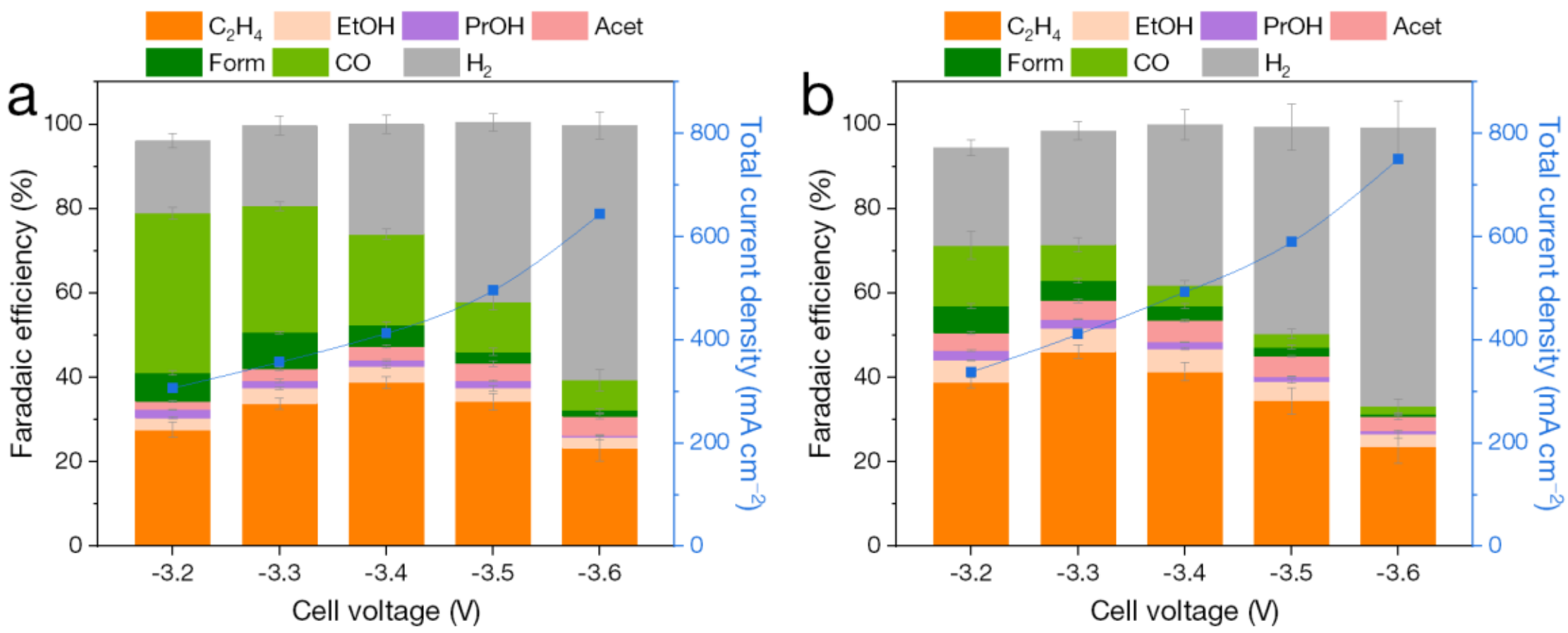

Figure S18. Product distribution during CO_2RR in MEA setup as a function of cell voltage for (a) Cu+K6 and (b) Cu/K6. Stacked bars show Faradaic efficiencies, and the blue line (right axis) denotes the corresponding total current density.

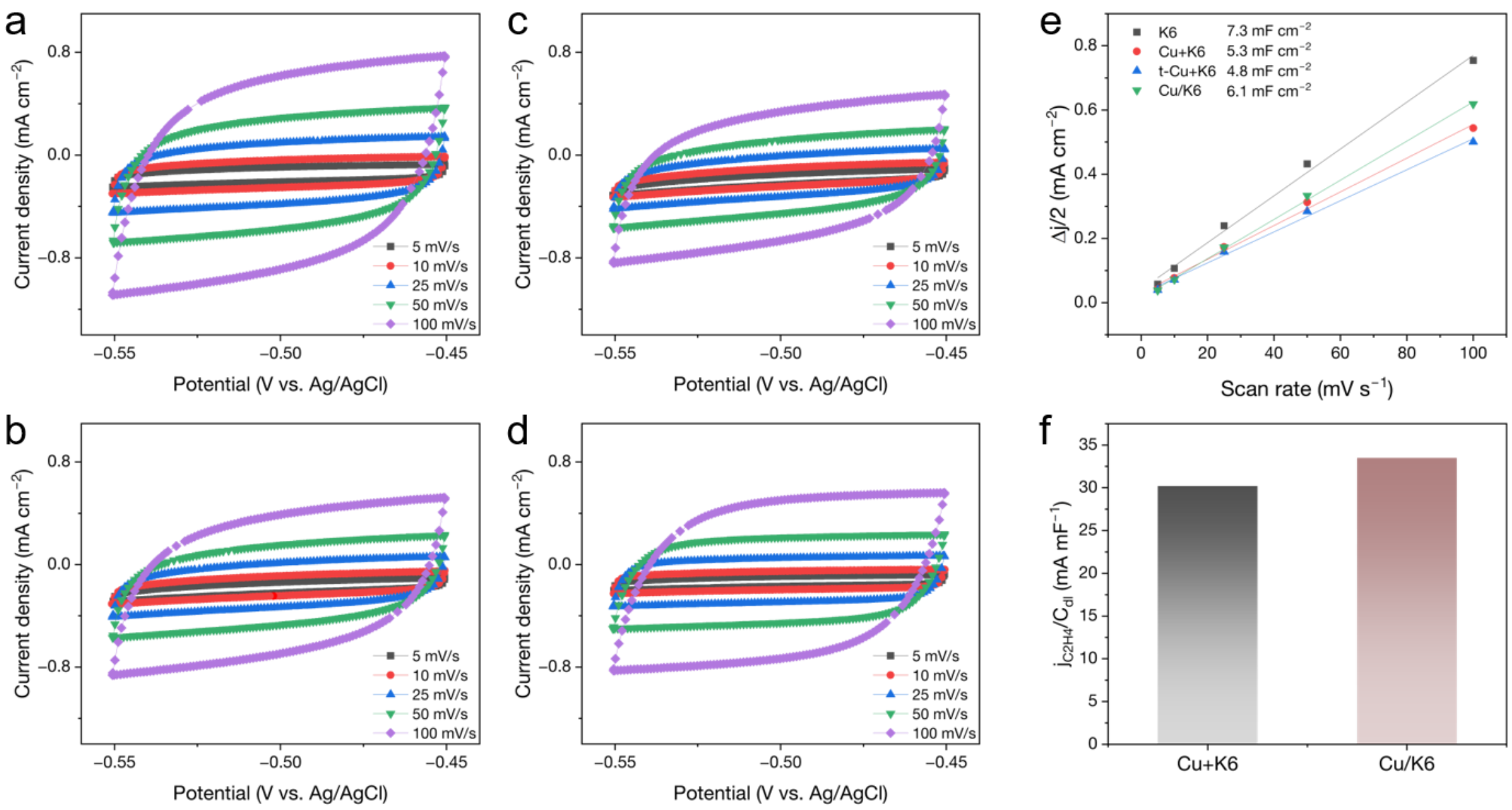

Figure S19. Cyclic voltammograms recorded in the non-Faradaic potential region for (a) K6, (b) Cu+K6, (c) t-Cu+K6, and (d) Cu/K6 electrodes. (e) Dependence of Δj/2 on scan rate to determine the double-layer capacitance (C_{dl}) from the linear slope. (f) C_{dl}-normalized C_2H_4 partial current densities of Cu+K6 and Cu/K6, calculated using their corresponding C_{dl} values and C_2H_4 partial current densities measured at cell voltage of −3.4 V in MEA.

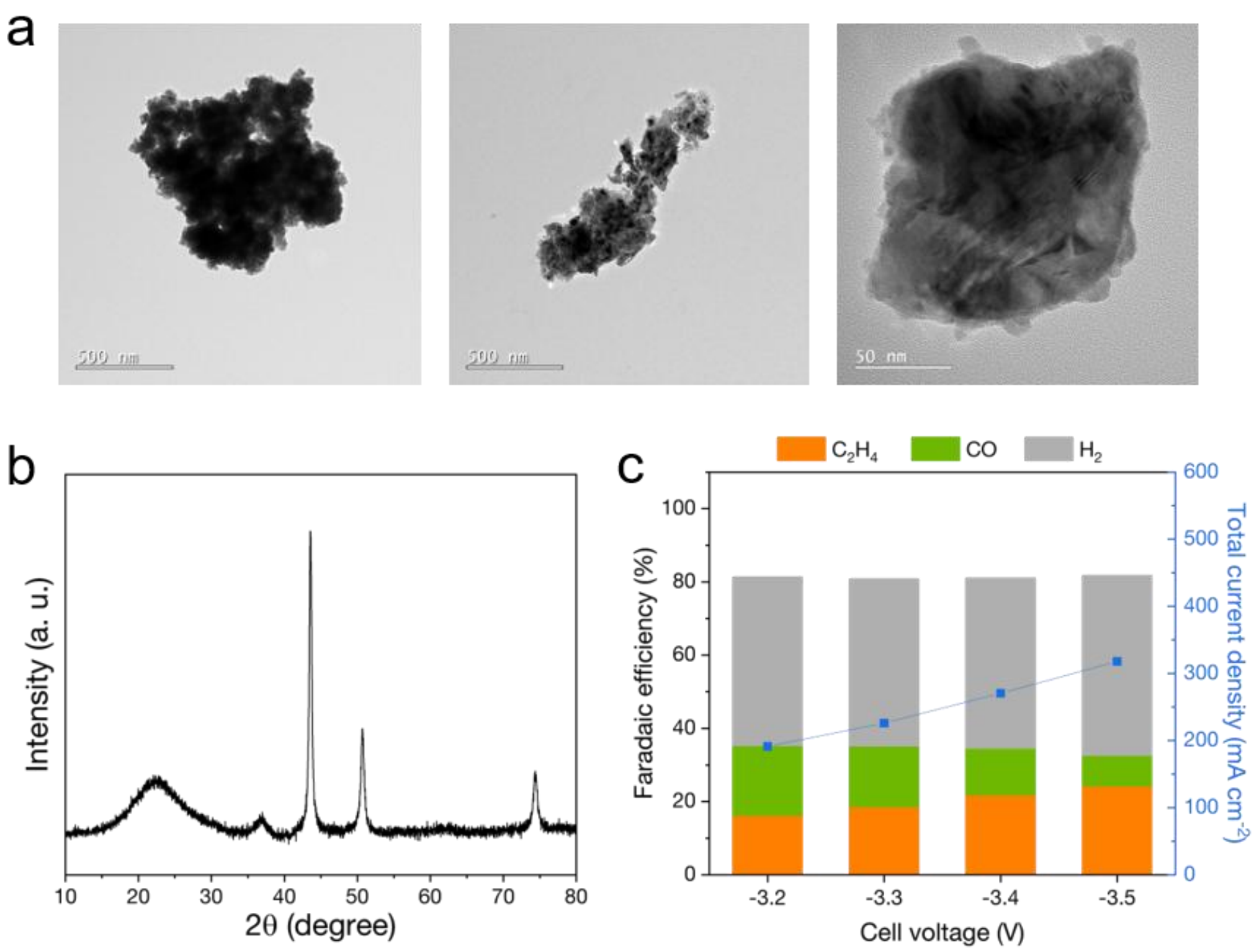

Figure S20. (a) TEM images of treated commercial Cu (t-Cu) at different magnifications. (b) XRD pattern of t-Cu. (c) Gas-product Faradaic efficiencies and total current density as a function of cell voltage for t-Cu+K6 cathodes in MEA.

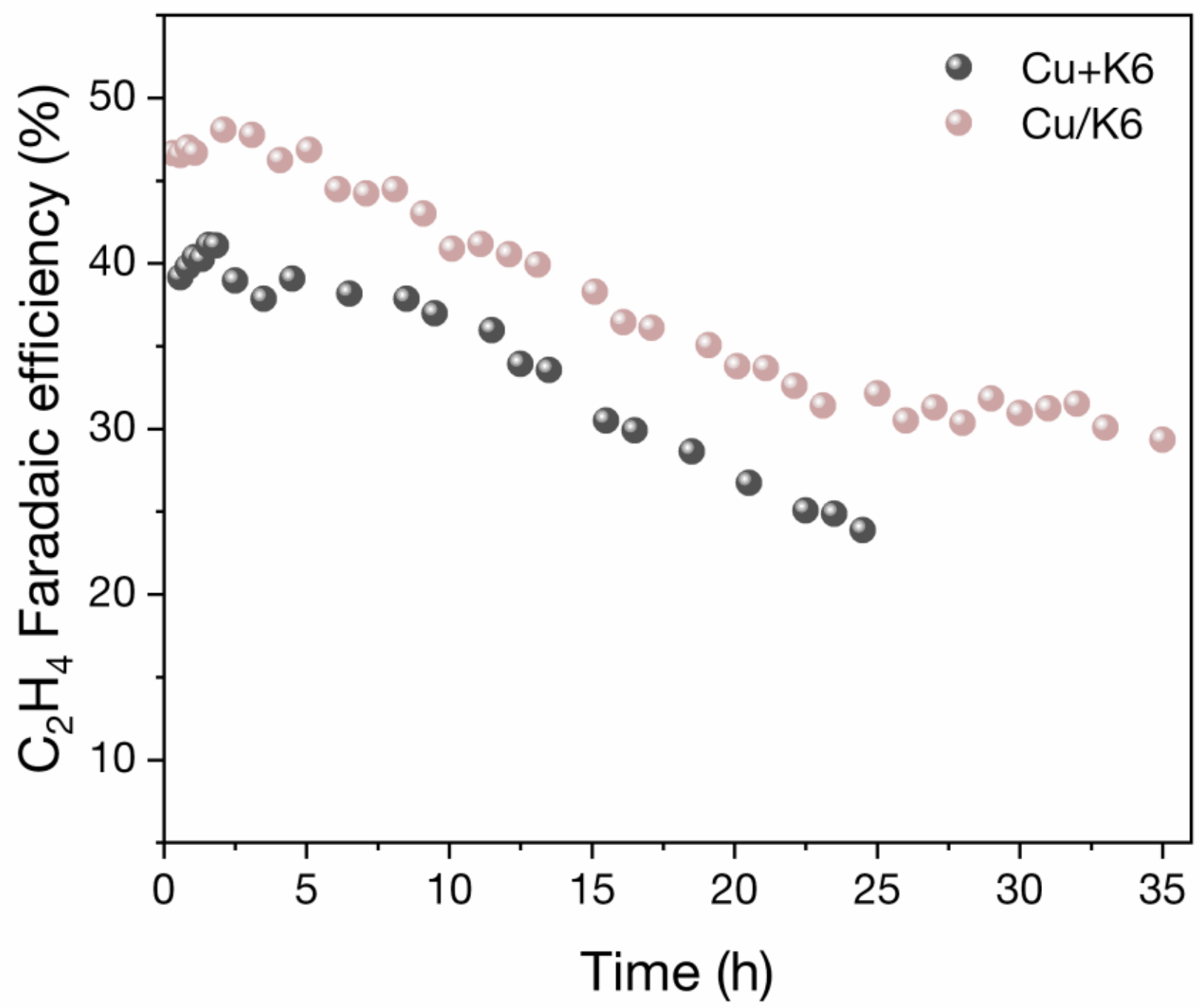

Figure S21. C_2H_4 Faradaic efficiency of Cu+K6 and Cu/K6 during extended CO_2RR operation at −3.3 V in MEA configuration.

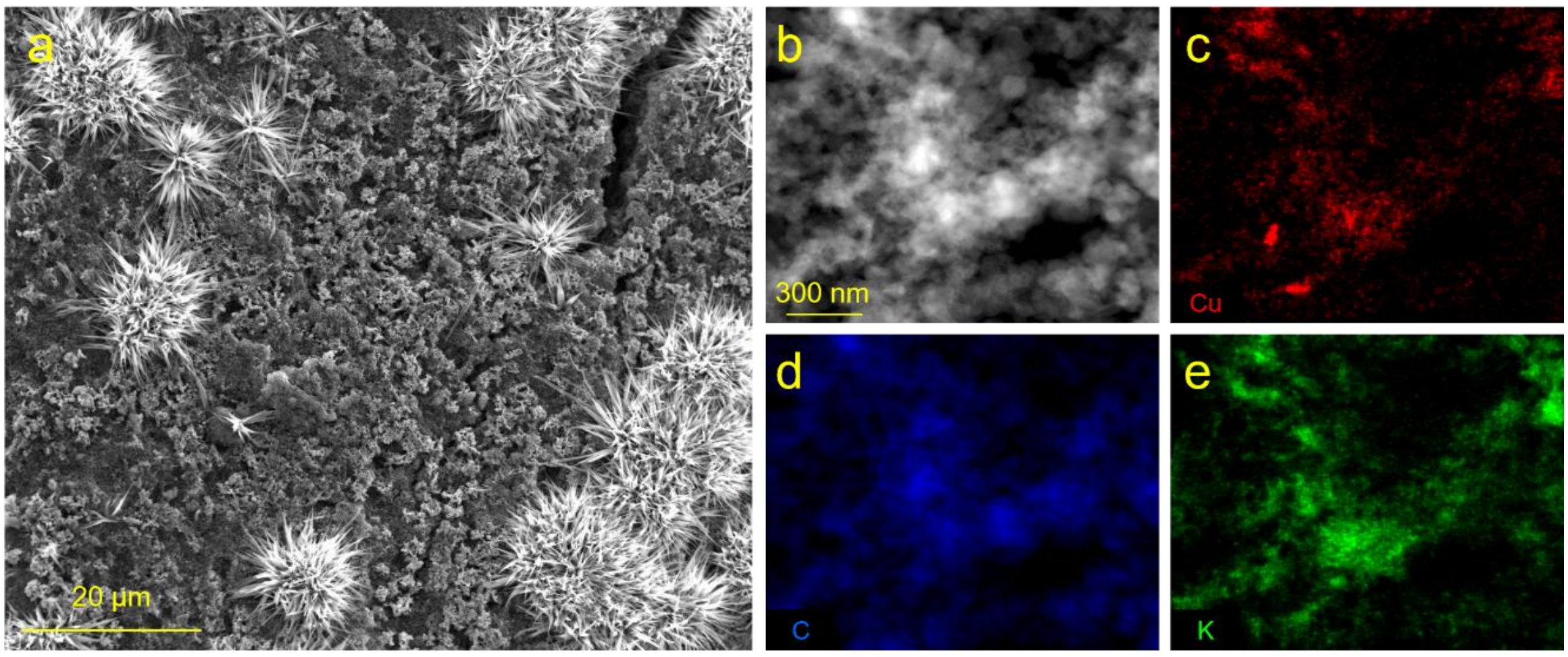

Figure S22. Post-stability morphological and elemental characterization of Cu/K6. (a) Ex situ SEM image of the electrode surface. (b) STEM image with corresponding elemental distributions of (c) Cu, (d) C, and (e) K.

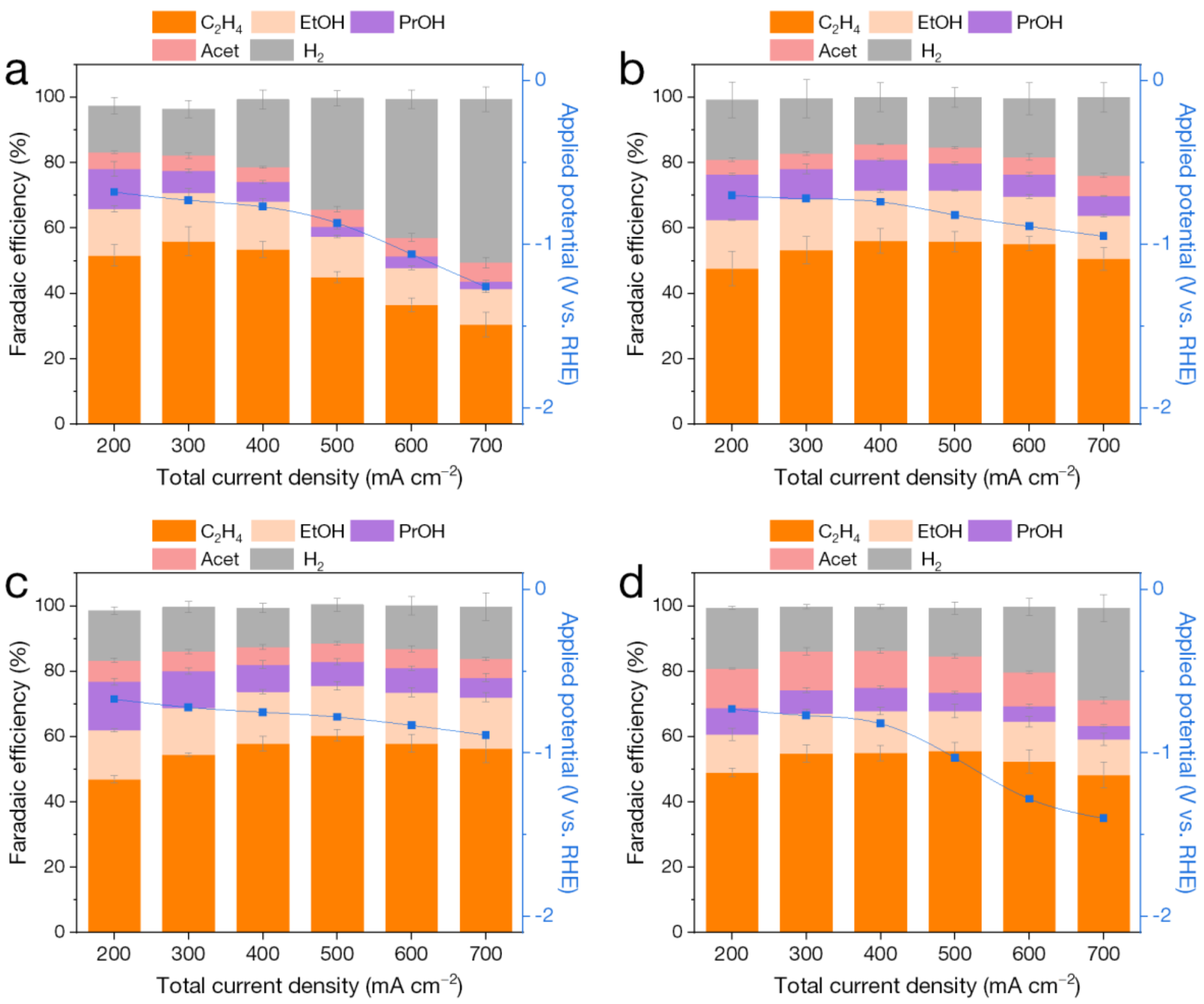

Figure S23. Product distribution during CORR in the flow cell setup as a function of total current density for (a) Cu/VC, (b) Cu/K3, (c) Cu/K6, and (d) Cu+K6. Stacked bars show Faradaic efficiencies, and the blue line (right axis) denotes the corresponding applied potential (V vs. RHE).

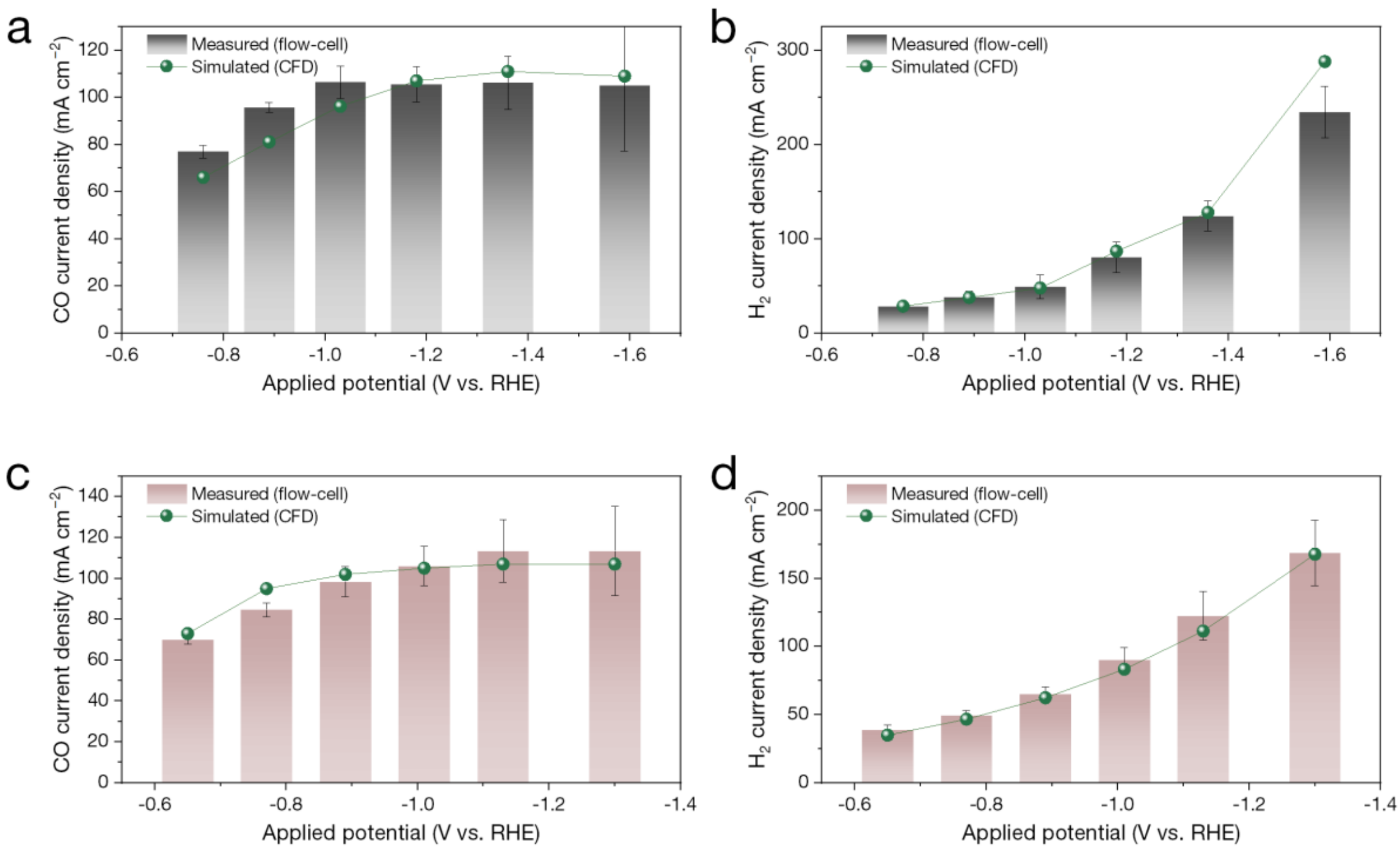

Figure S24. Comparison of experimentally measured and CFD-simulated partial current densities for (a, c) CO and (b, d) H_2. Results for (a, b) the physically mixed Cu+K6 cathode, and (c, d) the integrated Cu/K6 cathode.

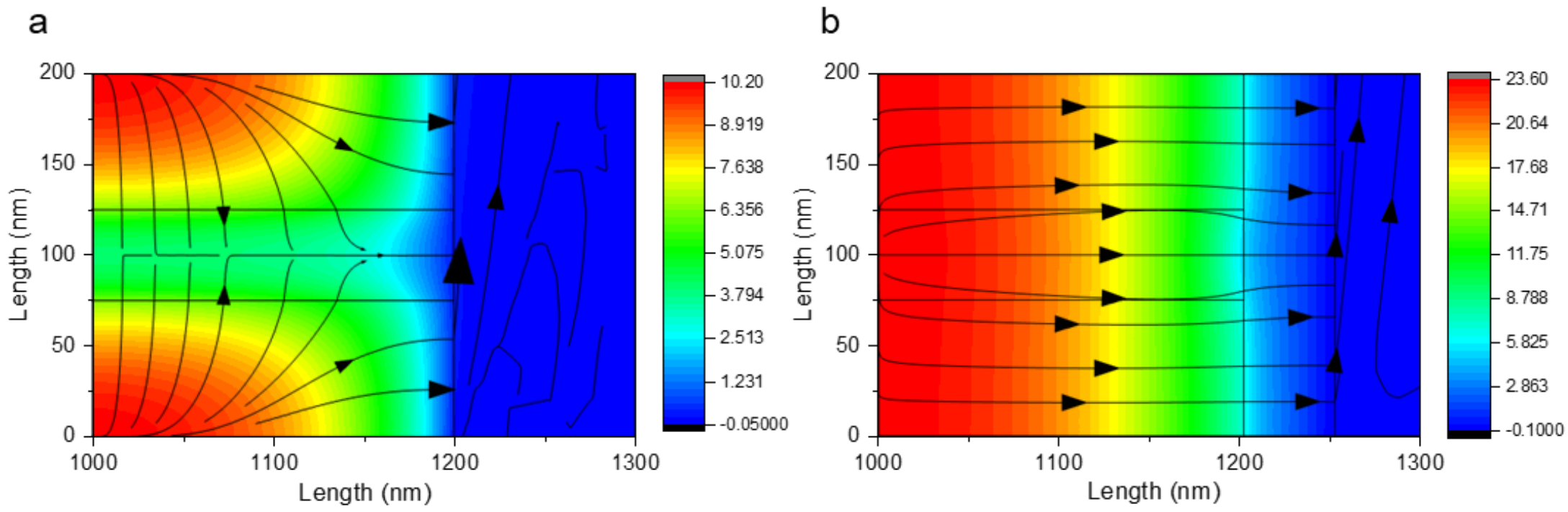

Figure S25. Comparison of CO_2 flux profiles in the catalyst layer and electrolyte flow channel. (a) Integrated catalyst model, highlighting the multi-directional CO_2 supply into the porous structure, and (b) physically mixed model, exhibiting limited CO_2 influx from the electrolyte flow channel.

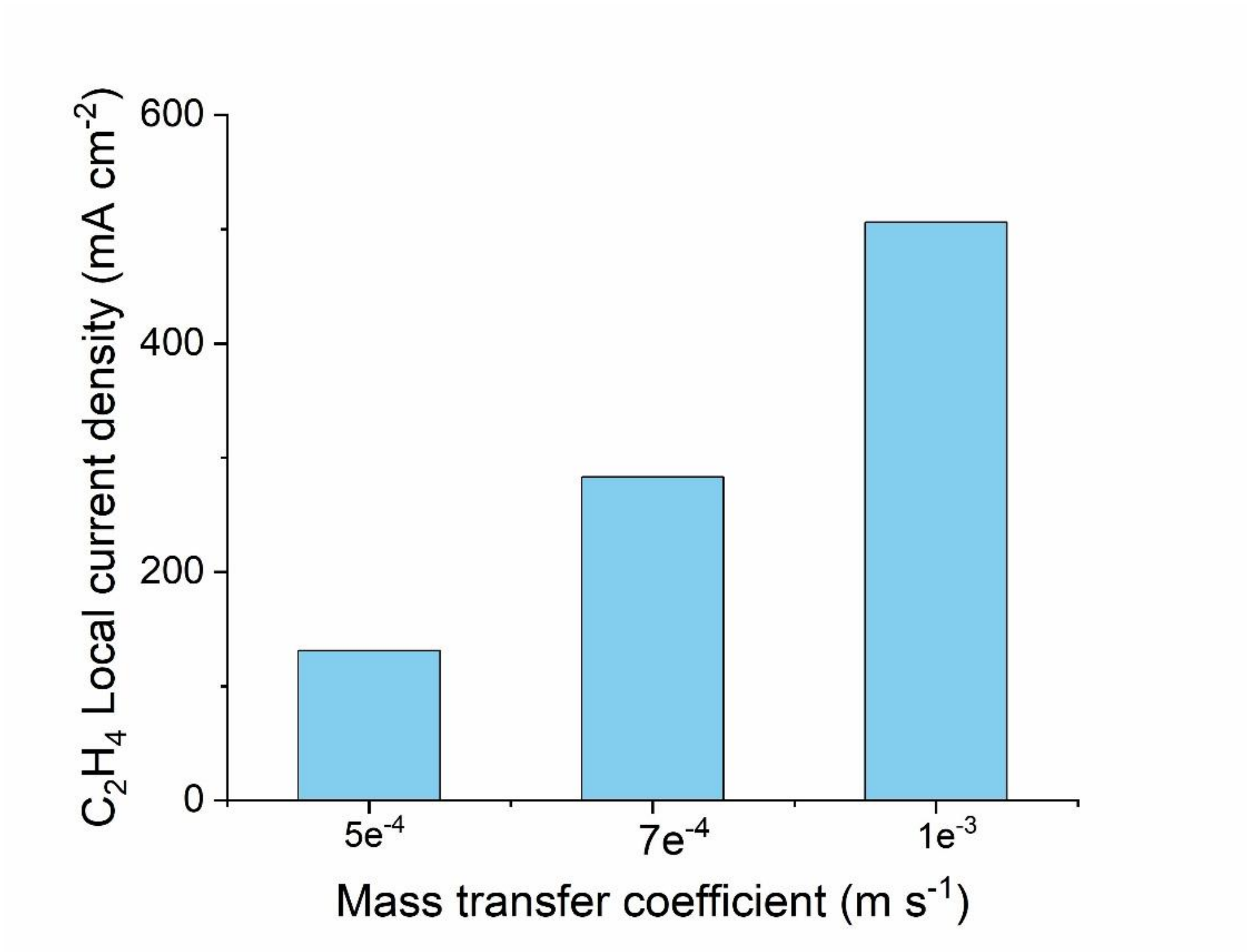

Figure S26. Sensitivity of C_2H_4 current density to the mass transfer coefficient.

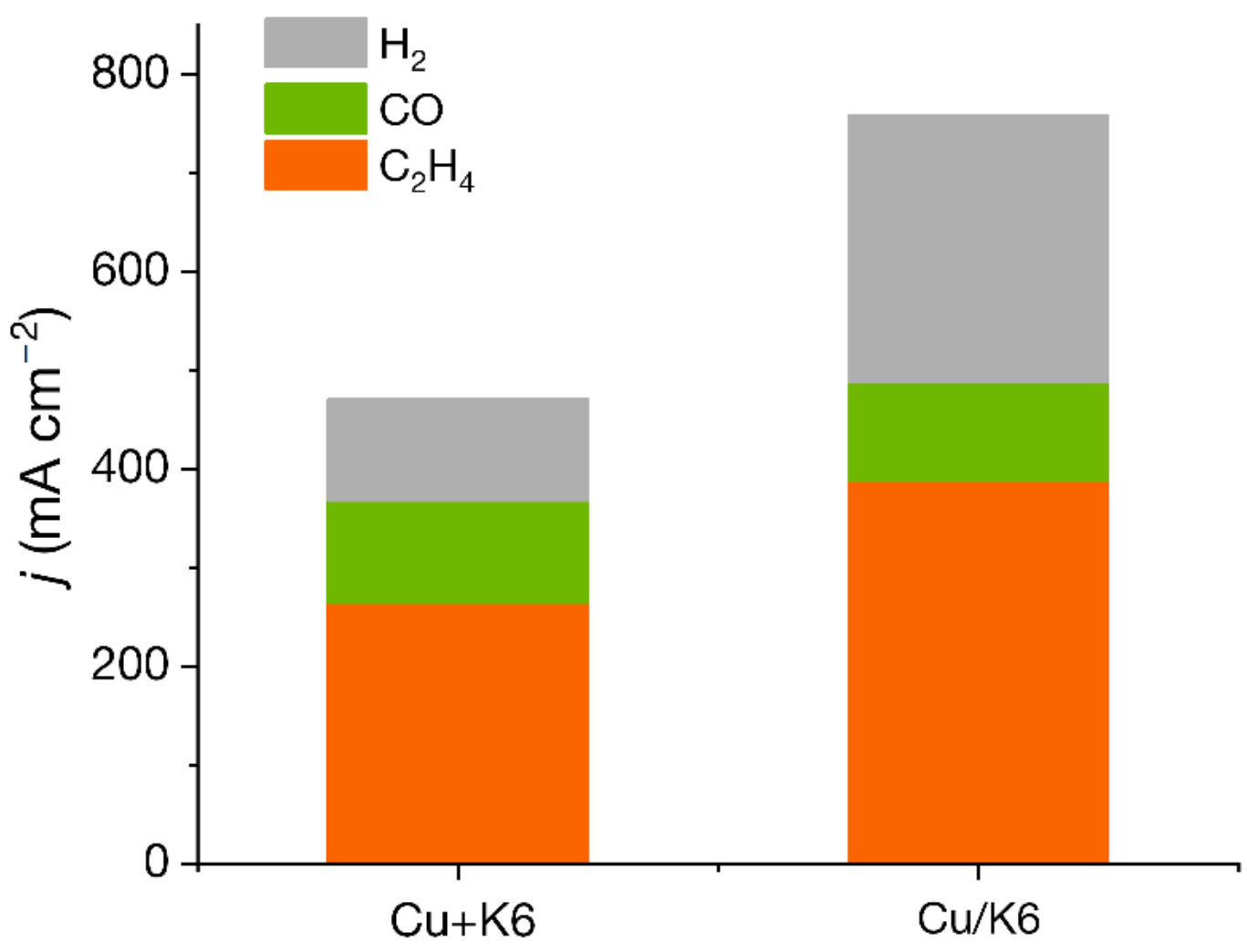

Figure S27. CFD-simulated local partial current densities for Cu+K6 and Cu/K6, showing the contributions from C_2H_4, CO, and H_2.

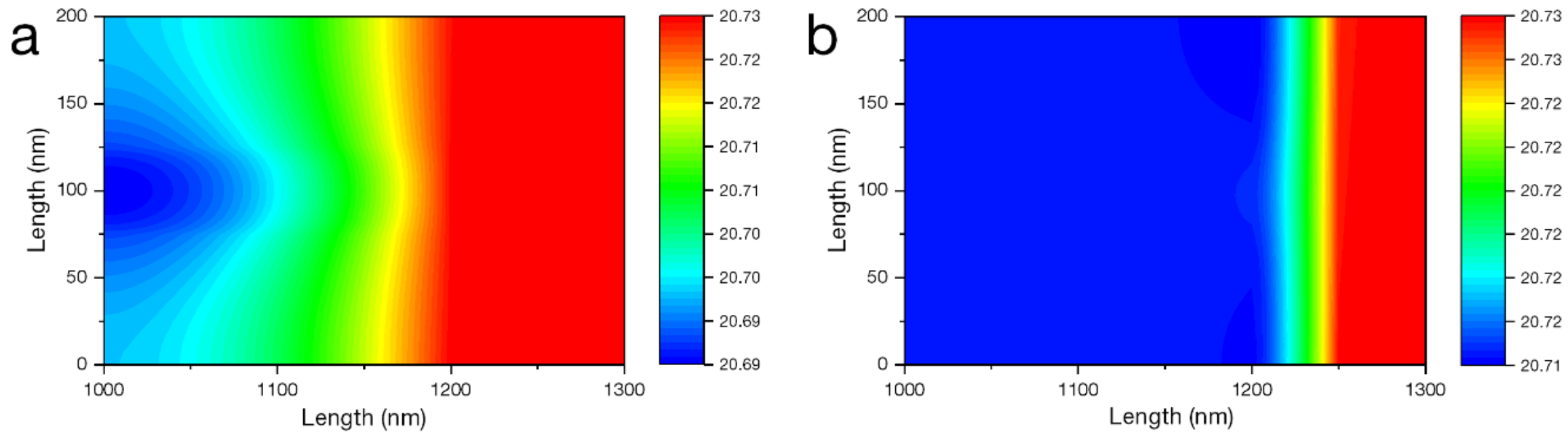

Figure S28. Water content in the catalyst layer and the electrolyte flow channel in (a) Cu/K6 and (b) Cu+K6.

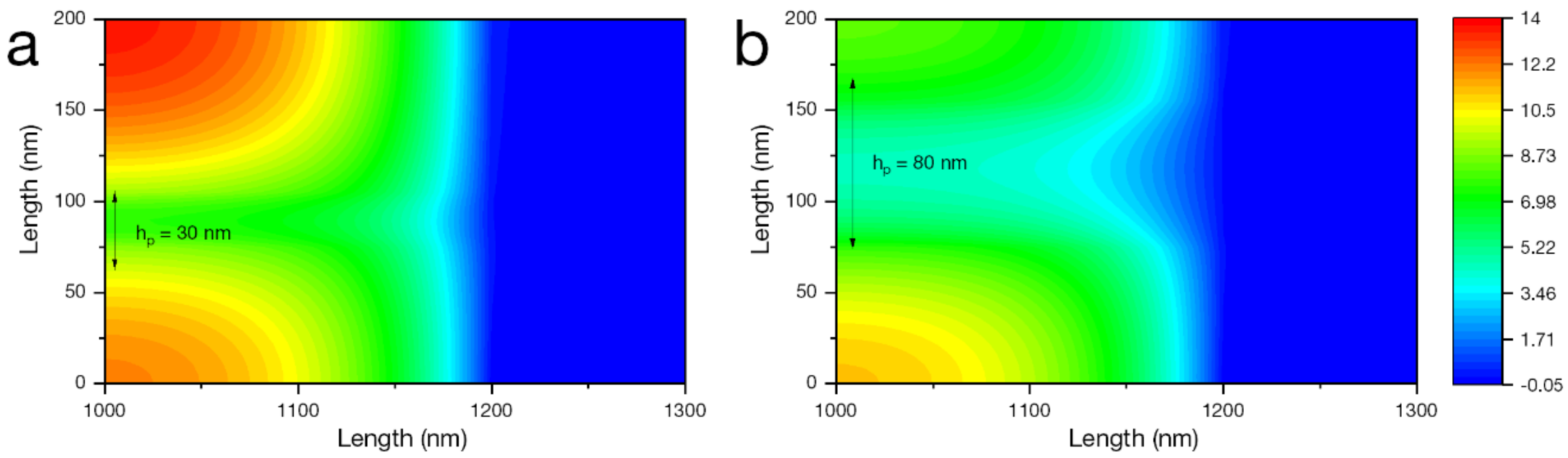

Figure S29. Impact of pore height (h_p), the pore diameter in the single-pore model, on local CO_2 concentration. (a) At h_p = 30 nm, (b) At h_p = 80 nm, CO_2 depletion is observed near the pore base due to the extended diffusion path (Simulation performed at a current density of 600 mA cm^{-2}).

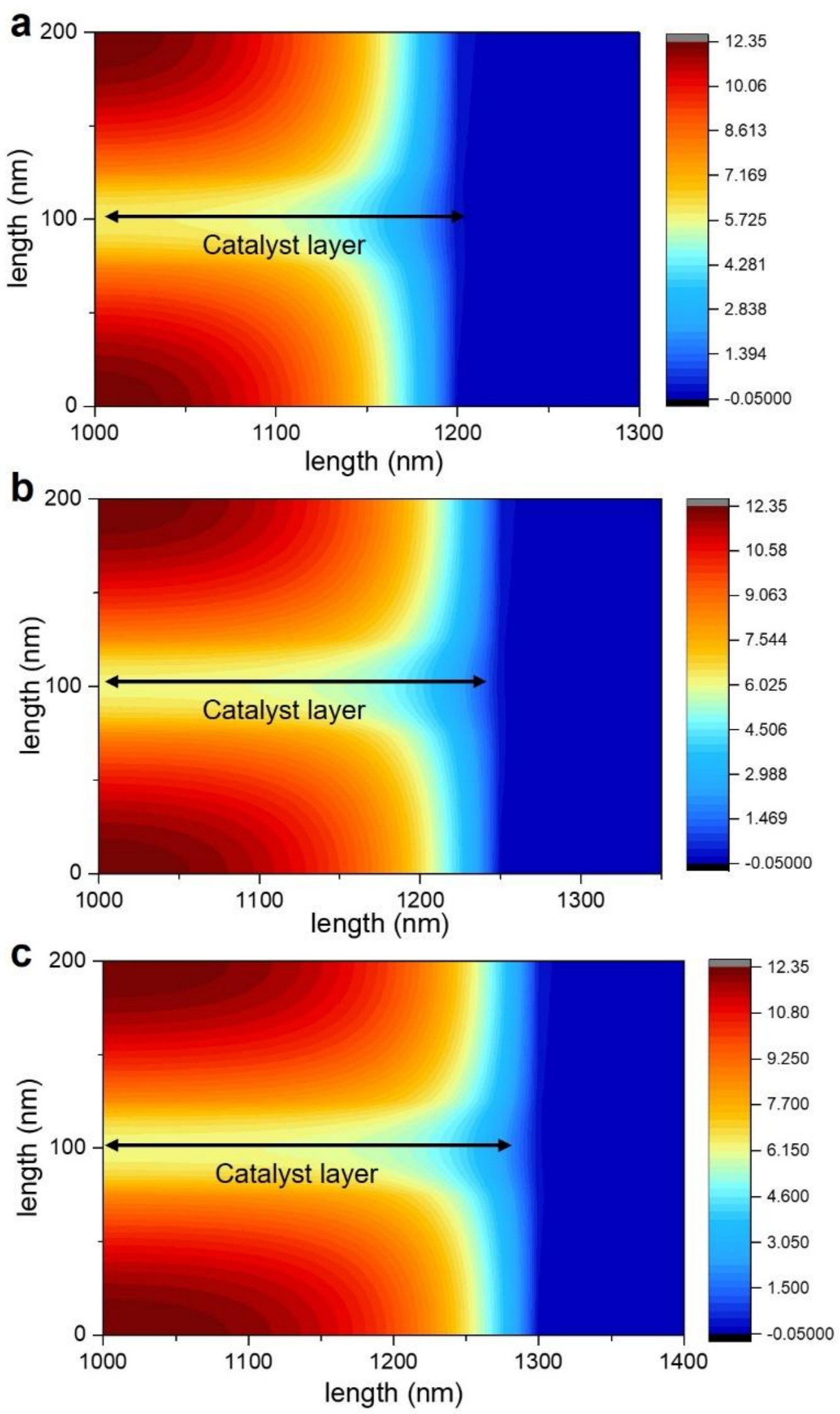

Figure S30. CFD-simulated CO_2 concentration in the single-pore model for pore depths of (a) 200 nm, (b) 250 nm, and (c) 300 nm. The depth was varied from the gas side toward the electrolyte, perpendicular to the pore height (h_p) in Figure S29. Because CO_2 enters the pore from the side opposite to the pore opening, a deeper pore shortens the gas-phase diffusion length and raises the CO_2 concentration. The baseline depth is 200 nm. Simulations were performed at 600 mA cm^{-2}. The color scale shows the CO_2 concentration in mol m^{-3}.

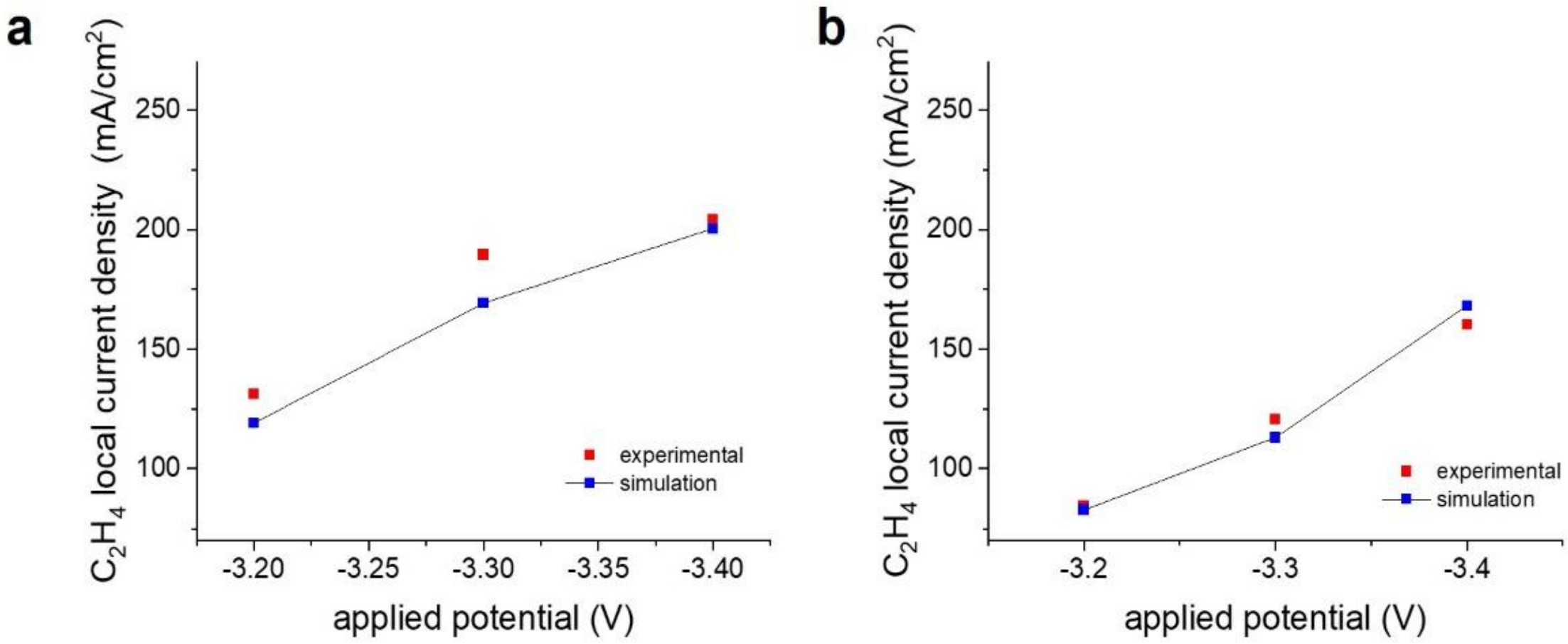

Figure S31. Comparison of experimentally measured and CFD-simulated C_2H_4 partial current densities (mA cm^{-2}) under an MEA-relevant operating condition for (a) Cu/K6 and (b) Cu+K6. The applied potential is the MEA cell voltage. The kinetic parameters calibrated with the flow-cell data were kept unchanged. The gas-side boundary was set to water vapor saturated at 30 °C, and the effective CO_2 diffusivity was adjusted to match only the measured total current density. The simulated values deviated from the measured values by at most 10.6% for Cu/K6 and 6.2% for Cu+K6.

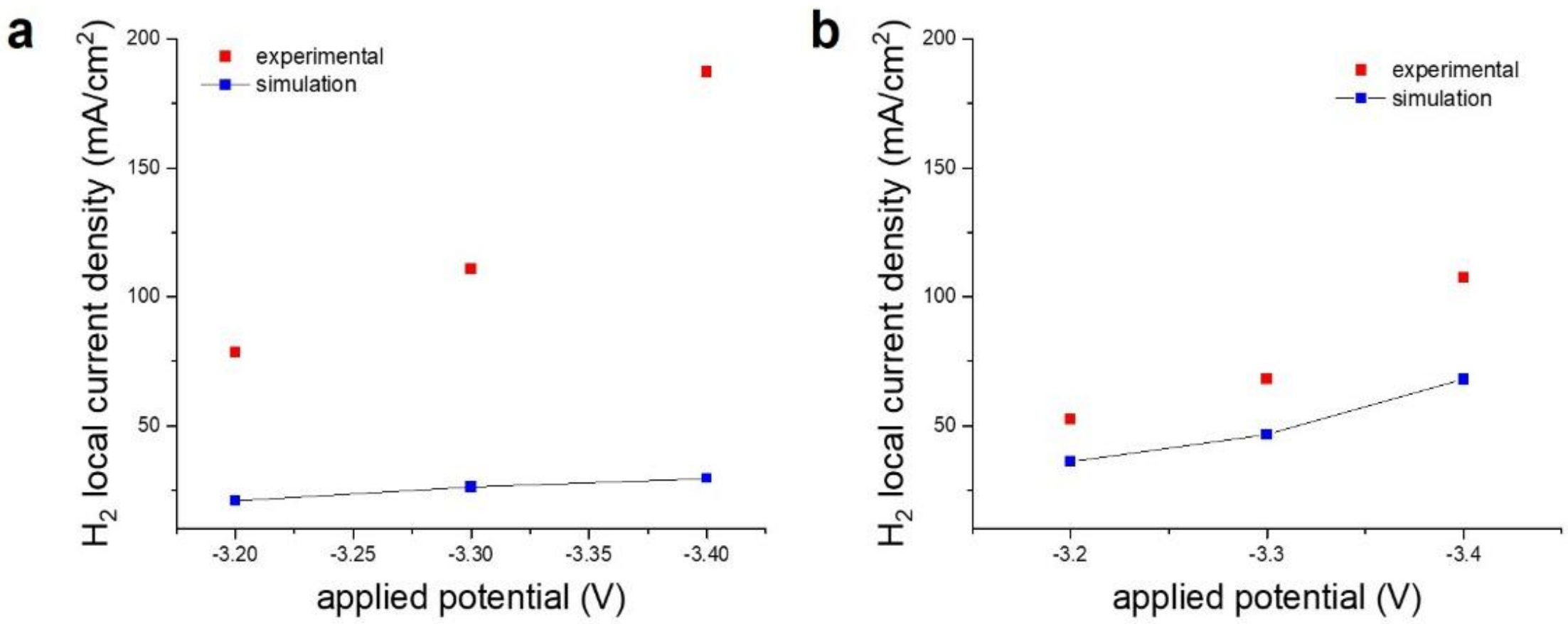

Figure S32. Comparison of experimentally measured and CFD-simulated H_2 partial current densities (mA cm^{-2}) under the same MEA-relevant operating condition as in Figure S31 for (a) Cu/K6 and (b) Cu+K6. The applied potential is the MEA cell voltage. The model underpredicted the H_2 partial current density for both cathodes.

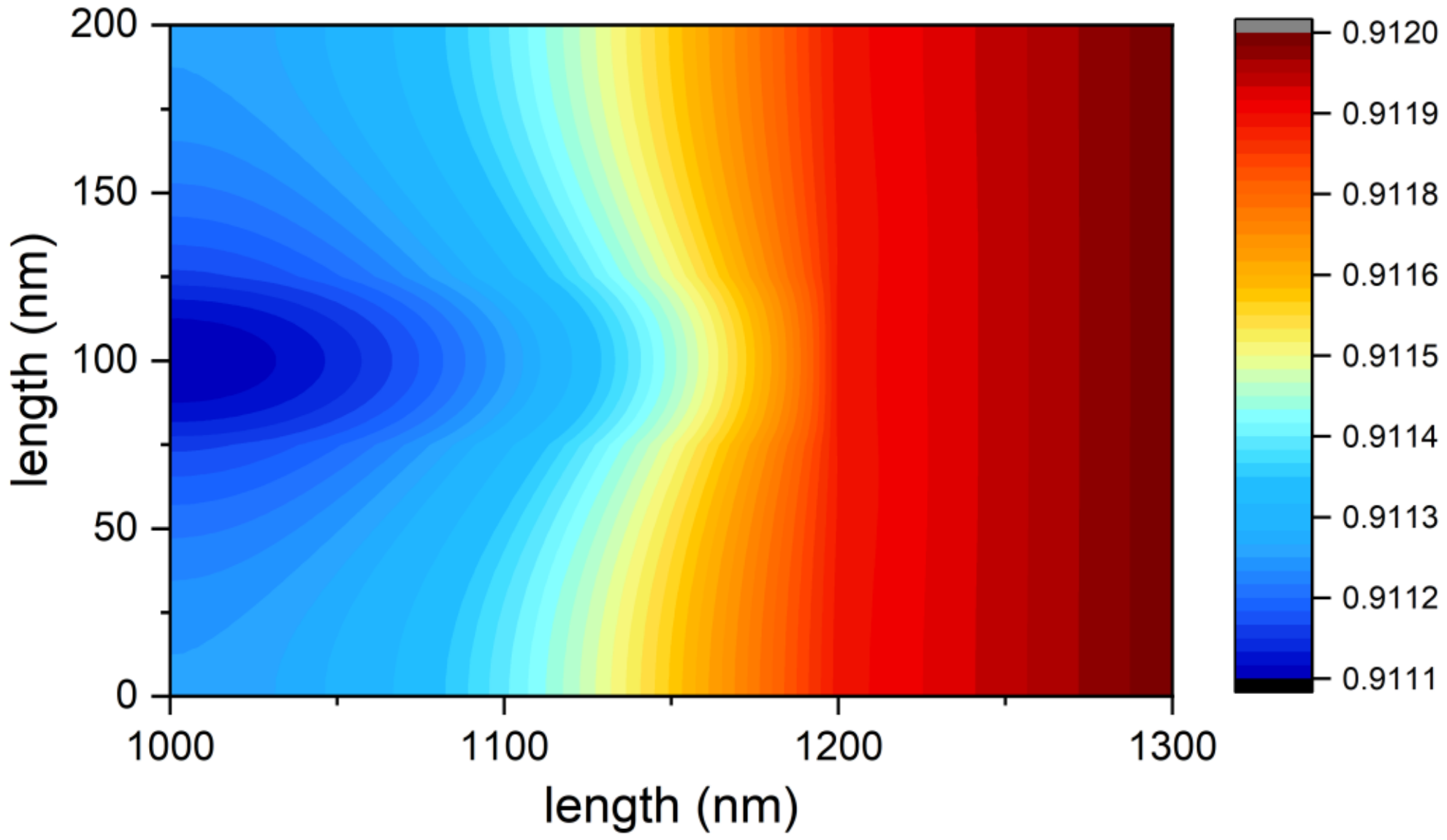

Figure S33. Contour map of the simulated water activity in the catalyst layer of Cu/K6 under the MEA-relevant operating condition used in Figures S31 and S32. The water activity ranged only from 0.9111 to 0.9120. The gas-side boundary was set to water vapor saturated at 30 °C. The water activity was not prescribed but obtained from the calculation.

Supplementary References

[S1] D.R. Lide, CRC Handbook of Chemistry and Physics: A Ready-Reference of Chemical and Physical Data, 85th ed., J. Am. Chem. Soc. 127 (2005) 4538–4542. https://doi.org/10.1021/ja041017a.

[S2] K.G. Schulz, U. Riebesell, B. Rost, S. Thoms, R. Zeebe, Determination of the rate constants for the carbon dioxide to bicarbonate inter-conversion in pH-buffered seawater systems, Mar. Chem. 100 (2006) 53–65. https://doi.org/10.1016/j.marchem.2005.11.001.

[S3] L.-C. Weng, A.T. Bell, A.Z. Weber, A systematic analysis of Cu-based membrane-electrode assemblies for CO_2 reduction through multiphysics simulation, Energy Environ. Sci. 13 (2020) 3592–3606. https://doi.org/10.1039/D0EE01604G.
